# Transcription of microRNAs is regulated by developmental signaling pathways and transcription factors

**DOI:** 10.3389/fcell.2024.1356589

**Published:** 2024-04-24

**Authors:** Malcolm Arnott, Nina Faye Sampilo, Jia L. Song

**Affiliations:** Department of Biological Sciences, University of Delaware, Newark, DE, United States

**Keywords:** post-transcriptional regulation, sea urchin, gene regulatory network, skeletogenesis, miR-1, miR-31

## Abstract

In early embryonic development, the cross-regulation of transcription factors and signaling pathways are critical in mediating developmental and physiological processes. Additionally, many studies have shown the importance of post-transcriptional regulation of signaling and network components mediated by microRNAs (miRNAs); however, how miRNAs are transcriptionally regulated is poorly understood. miRNAs are critical fine-tuners of many biological processes and their dysregulation leads to a variety of diseases and developmental defects. Previously, we have shown that miRNAs are dynamically expressed throughout sea urchin development, suggesting that miRNAs are likely to be under transcriptional regulation. Here, we used pharmacological inhibitors, genetic constructs, and loss-of-function reagents to assess the impact of key signaling pathways (Wnt, Nodal, MAPK, Sonic Hedgehog, Delta/Notch, VEGF, and BMP) and transcription factors (Alx1, Ets1/2, and Tbr) on the transcript levels of the evolutionarily conserved miR-1, miR-31, miR-92 and miR-124; the invertebrate-specific miR-71; and the echinoderm-specific miR-2002, miR-2007, and miR-2012. We also used computational methods to identify potential transcription factor binding sites of these miRNAs. Lists of binding motifs for transcription factors (TFs) were acquired from the MEME-Suite Motif Database and used as inputs for the algorithm FIMO (Find Individual Motif Occurrences), which detects short nucleotide motifs within larger sequences. Based on experimental data on miRNA expression in conjunction with bioinformatic predictions, we propose that the transcription factors Tbr, Alx1, and Ets1 regulate *Sp*miR-1, *Sp*miR-31, and *Sp*miR-71, respectively. We additionally observed significant effects on miRNA levels as a result of perturbations to Wnt, Nodal, MAPK, and Sonic Hedgehog signaling pathways, while no significant change on miRNA levels were observed with perturbations to Delta/Notch, VEGF, or BMP signaling pathways. Overall, this study provides insights into the transcriptional regulation of miRNAs by signaling pathways and transcription factors and contribute to our overall understanding of the genetic regulation of developmental processes.

## 1 Introduction

microRNAs (miRNAs) are a class of small non-coding RNAs that are key mediators of post-transcriptional gene regulation (Lee et al., 1993; Wightman et al., 1993). Mature miRNA sequences have an average of 22 nucleotides, many of which are conserved among metazoans (Wheeler et al., 2009; Fromm et al., 2015; Bartel, 2018). Evidence in the past three decades have demonstrated that they are highly evolutionarily conserved in performing critical regulatory roles in fine-tuning of gene expression to modulate cell proliferation, cell differentiation, and the physiological functions of cells and embryos (Brennecke et al., 2003; Xu et al., 2003; Chen et al., 2004; Wienholds et al., 2005; Robinson, 2009; Wahid et al., 2010). Studies have shown that miRNAs are essential for early embryogenesis, where depletion of global miRNAs with loss-of-function of key miRNA biogenesis enzymes, Drosha and Dicer, resulted in severe developmental defects and embryonic lethality (Bernstein et al., 2003; Giraldez et al., 2005; Song et al., 2012; Saurat et al., 2013). We previously found that gastrulation failure and embryonic lethality induced by Drosha and/or Dicer morpholino antisense oligonucleotide (MASO)-injection in sea urchin embryos were rescued by co-injection with four of the most abundantly expressed miRNAs (*Sp*miR-1, *Sp*miR-31, *Sp*miR-71 and *Sp*miR-2012) (Song et al., 2012). Interestingly, highly expressed miRNAs tend to be functionally important and evolutionarily conserved (Liang and Li, 2009). While extensive progress has been made in understanding the importance of miRNAs as post-transcriptional regulators, relatively little is known about transcriptional regulation of miRNAs.

To examine how miRNAs are transcriptionally regulated in early embryonic development, we use the purple sea urchin *Strongylocentrotus purpuratus* as our model organism. Sea urchins are closely related to chordates with about 70% of sea urchin genes having a human counterpart (Davidson et al., 2002; 2020; Davidson, 2006; Sodergren et al., 2006). We take advantage of their well-characterized signaling pathways and gene regulatory networks (GRNs), their high fecundity, and comparatively rapid and predictable early developmental life cycle (Sodergren et al., 2006; McClay, 2011). The sea urchin has only ∼50 annotated miRNAs, which is in contrast to humans that have 519 miRNAs (Hinman et al., 2003; Wheeler et al., 2009; Song et al., 2012; Bartel, 2018). About 80% of the miRNAs found in the sea urchin genome are also present in chordates, and many are found in protostomes as well (Wheeler et al., 2009; Song et al., 2012). Using a combination of pharmacological inhibitors, genetic constructs, and morpholino antisense oligonucleotides (MASOs), we examined the regulatory impact of key signaling pathways (Wnt, Nodal, MAPK, Sonic Hedgehog (SHH), Delta/Notch, VEGF, and BMP), in addition to transcription factors (Alx1, Ets1/2, and Tbr) on *Sp*miR-1, *Sp*miR-31, *Sp*miR-92, *Sp*miR-71, *Sp*miR-124, *Sp*miR-2002, *Sp*miR-2007, and *Sp*miR-2012. Of the set of miRNAs examined, miR-1, miR-31, miR-92 and miR-124 are highly evolutionarily conserved throughout metazoans (Wheeler et al., 2009; Takane et al., 2010; Concepcion et al., 2012; Song et al., 2012; Bartel, 2018). miR-71 is conserved across insects and invertebrates (Marco et al., 2010; de Souza Gomes et al., 2013; Pérez et al., 2019); miR-2012 has only been annotated in the sea urchin, sea star, acorn worm, and *Xenoturbella* (Philippe et al., 2011; Song et al., 2012); and miR-2002 and miR-2007 are sea urchin specific miRNAs originating in the clade Eleutherozoa (Wheeler et al., 2009). Additionally, computational predictions were performed to identify TF binding motifs upstream of miRNA genomic loci. We examined if these predicted TFs were downstream of the signaling pathways that we tested to impact the level of miRNAs.

The sea urchin utilizes highly conserved signaling pathways to regulate development, including Wnt, Nodal, MAPK, SHH, Delta/Notch, VEGF, BMP signaling pathways. The broader Wnt signaling pathway can be separated into three main branches: canonical Wnt (cWnt), non-canonical Wnt/Planar Cell Polarity (ncWnt/PCP) pathway and the ncWnt/Ca^2+^ pathway (Komiya and Habas, 2008). The cWnt branch, which uses β-catenin as the key transducer, is critical for anterior/posterior primary body axis formation, cell differentiation and germ layer specification (Wikramanayake et al., 2004; Kumburegama and Wikramanayake, 2008). While cWnt is involved in anterior-posterior body axis, Nodal signaling pathway is involved in ventral-dorsal body axis formation (Duboc et al., 2005). In fact, cWnt is involved in activating Nodal signaling, and together they function antagonistically to set up the body plan of the embryo (Wei et al., 2012). MAP kinases phosphorylate Yan/Tel transcriptional repressor, which restricts expression of *Nodal* (Molina et al., 2018). Yan/Tel morphants led to expanded expression of Nodal and a radialized embryo with disrupted dorsal-ventral axis. Nodal is involved in specifying the ventral ectoderm. Activated by Nodal, BMP signaling is required for specification of the dorsal/ventral and left/right (L/R) body axes and responsible for maintaining the dorsal gene expression (Duboc et al., 2004; 2005; Furtado et al., 2008). Nodal and BMP signaling pathways work together to set up the dorsal-ventral body axis, as well as repressing neural ciliary band gene fates (Saudemont et al., 2010). The Delta/Notch signaling pathway in echinoderms is involved in segregation of the endomesoderm and specification of the secondary mesenchyme cells (SMCs) (Yoon and Gaiano, 2005; Kopan, 2012; Yaguchi et al., 2012; Yankura et al., 2013; Burke et al., 2014; Jiao et al., 2017; Siebel and Lendahl, 2017; McClay et al., 2018). SHH is involved in muscle fiber organization and patterning of the mesoderm, as well as mediating Nodal’s patterning of the L/R axis (Johnson et al., 1994; Nelson et al., 1996; Walton et al., 2009; Low and Sauvage, 2010; Warner et al., 2016). VEGF signaling is involved in directed migration of skeletogenic cells (Duloquin et al., 2007; Adomako-Ankomah and Ettensohn, 2013; Adomako-Ankomah and Ettensohn, 2014).

We have previously found *Sp*miR-1 and *Sp*miR-31 to regulate sea urchin skeletogenesis (Stepicheva and Song, 2015; Sampilo and Song, 2024). The Wnt and VEGF signaling pathways we examined here have also been shown to play important roles in skeletal development (Croce et al., 2006; Adomako-Ankomah and Ettensohn, 2013; McIntyre et al., 2013; Morgulis et al., 2021). Sea urchin skeletogenesis may be analogous to vertebrate angiogenesis and vascularization (Morgulis et al., 2019; Gildor et al., 2021). Both processes use a common set of TFs (Ets1/2, Erg, Hex, Tel, and FoxO) and signaling pathways (VEGF Nodal, BMP, Delta/Notch, and Angiopoetin). Transcription factors and signaling pathways important for vascularization are expressed and utilized in the sea urchin skeletogenic cells (primary mesenchyme cells; PMCs) at the time of migration and patterning, and in skeletal formation (Duboc et al., 2004; Oliveri et al., 2008; Morgulis et al., 2019). Prior work has shown that Ets1/2, Tbr, and Alx1 are all key regulators of sea urchin skeletogenesis (Fuchikami et al., 2002; Ettensohn et al., 2003; Oliveri et al., 2008). Of these, we found *Sp*miR-1 to suppress reporters containing 3′UTRs of *Ets1/2* and *Tbr* (Sampilo and Song, 2024) and *Sp*miR-31 to suppress reporters containing 3′UTR of *Alx1* and *VegfR7* (Stepicheva and Song, 2015). Moreover, among the deuterostomes, only echinoderms and vertebrates produce extensive skeletons, while other bilaterians such as hemichordates and tunicates do not form extensive skeletons (Murdock, 2020; Ben-Tabou de-Leon, 2022). Conserved TFs involved in skeletal development include Ets1 and Alx1. Ets1 is a TF which regulates skeletogenesis in vertebrates by promoting preosteoblast proliferation; in echinoderms, Ets1 is involved in skeletogenic cell specification (Kurokawa et al., 1999; Raouf and Seth, 2000; Consales and Arnone, 2002; Ettensohn et al., 2003; Oliveri et al., 2008; Sharma and Ettensohn, 2010; Damle and Davidson, 2011). Alx1 is involved in regulating craniofacial structures in vertebrates (Zhao et al., 1996; Lyons et al., 2016; Garg et al., 2017; Pini et al., 2020). In the sea urchin, it is the main driver of skeletogenic specification (Ettensohn et al., 2003; McCauley et al., 2012; Erkenbrack and Davidson, 2015; Koga et al., 2016; Khor and Ettensohn, 2017).

miRNAs in general are a necessary component of the developmental program (Bernstein et al., 2003; Giraldez et al., 2005; Song et al., 2012; Saurat et al., 2013). For example, miR-1, known as a myomiR, regulates heart formation in vertebrates (Mansfield et al., 2004; Sokol and Ambros, 2005; Wienholds et al., 2005; Zhao et al., 2005; 2007; McCarthy, 2011). In the sea urchin, we found *Sp*miR-1 to regulate circumpharygeal muscle fibers and skeletogenesis (Sampilo and Song, 2024). Additionally, previous work has shown that in vertebrates, miR-31 regulates osteoblast proliferation and myogenesis (Cacchiarelli et al., 2011; Crist et al., 2012; Baglìo et al., 2013; Deng et al., 2013; Stepicheva and Song, 2016). In the sea urchin embryo, inhibition of *Sp*miR-31 or blockage of *Sp*miR-31’s suppression of *Alx1* results in skeletogenic cell patterning and spicule formation defects (Stepicheva and Song, 2015). While it is less well studied in echinoderms and vertebrates, work in *C. elegans* has shown miR-71 to be involved in L/R axis specification and aging, with additional work showing that it is necessary for survival of primary cells in the parasitic tapeworm *Echinococcus multilocularis*, and oogenesis in the migratory locust *Locusta migratoria* (Hsieh et al., 2012; Lucanic et al., 2013; Pérez et al., 2019; Song et al., 2019)*.* In both the sea urchin and vertebrates, miR-124 has the conserved function of regulating neuronal development and neurogenesis; in addition, in the sea urchin, *Sp*miR-124 is involved in specification of immune cells by regulating Delta/Notch and Nodal signaling pathways (Liu et al., 2011; Jiao et al., 2017; Konrad and Song, 2022; Konrad et al., 2023). Based on the myriad roles of miRNAs in the developmental programs of various species, it is clear that investigation into the transcriptional regulation of miRNAs can provide valuable insights into the regulation of development.

Results from this study revealed that disruption of the signaling pathways, including Wnt, Nodal, MAPK, and SHH, resulted in expression level changes of several miRNAs, while perturbation of Delta/Notch, VEGF, and BMP signaling pathways did not yield significant changes of these miRNAs. Interestingly, one of the TFs identified as a potential regulator of miR-31 transcription, Alx1, has been previously identified as a target of *Sp*miR-31 (Stepicheva and Song, 2015). Similarly, we have previously identified *Sp*miR-1 to regulate *Tbr*; in this study, *Tbr* loss-of-function leads to significant decrease of *Sp*miR-1 (Figure 3) (Sampilo and Song, 2024). Our results identify specific signaling pathways and TFs that likely regulate the transcription of miRNAs. We also discover cross-regulation amongst miRNAs, TFs, and signaling pathways as important regulators of embryonic development.

## 2 Materials and methods

### 2.1 Animals

Adult purple sea urchins, *S. purpuratus* (*Sp*), were obtained from Point Loma Marine Invertebrate Lab, (Lakeside, CA) and Marinus Scientific, LLC (Long Beach, CA). Adult males and females were injected with 0.5 M KCl intracoelomically to obtain sperm and eggs. Filtered natural seawater (FSW) (collected from Indian River Inlet; University of Delaware) or artificial seawater (ASW) made from Instant Ocean^©^ was used for embryo cultures incubated at 15°C.

### 2.2 Pharmacological inhibitors

Pharmacological inhibitors against signaling pathways were tested at various concentrations and time points to establish ideal conditions that produce expected published phenotypes without toxicity (Supplementary Table S1). Axitinib (Duloquin et al., 2007), Bisindolylmaleimide-I (Toullec et al., 1991; Gekeler et al., 1996), C59 (Cui et al., 2014), Cyclopamine (Batsaikhan et al., 2014), and SP600125 (Bennett et al., 2001) were purchased from Selleckchem (Houston, TX, Catalog numbers: S1005, S7208, S7037, S1146, S1460, respectively). A-83-01 (Tojo et al., 2005) and SB431542 (Inman et al., 2002; Duboc et al., 2005) were purchased from Tocris Bioscience (Minneapolis, MN, Catalog number 2930 and 1614, respectively). U0126 (Kumano and Foltz, 2003; Rottinger et al., 2004) and Y-27632 (Beane et al., 2006) were purchased from Calbiochem (San Diego, CA, Catalog numbers 662005 and 688000, respectively). Dorsomorphin was purchased from Sigma Aldrich (P5499-5mg) (Luo and Su, 2012). DAPT (Materna and Davidson, 2012) and Omeprazole (Bessodes et al., 2012) were purchased from Calbiochem (CAS-208255-80-5 and 0104-100mg, respectively). ML141 (Surviladze et al., 2010) was purchased from Cytoskeleton, Inc. (Denver, CO, Cat# BK034). Inhibitors were reconstituted in DMSO. Fertilization envelopes were removed by fertilizing eggs in FSW with 1mM 3-AT (Millipore Sigma, St. Louis, MO; A8056-25G) on protamine sulfate-coated dishes and gently detaching them with a Pasteur pipette to ensure drug penetration. Embryos were cultured in control DMSO, or drug-treated FSW at 2-cell stage until blastula stage, followed by subsequent three washes with FSW prior to collection. Effective concentrations for treatment were based on prior studies.

### 2.3 Microinjections

Microinjections were performed as previously described (Cheers and Ettensohn, 2004; Stepicheva and Song, 2014) with modifications. All injection solutions were prepared in a 2.5 µL solution consisting of 0.5 µL of 100% glycerol and 0.5 µL of 2 mg/mL 10,000 MW neutral non-fixable Texas Red dextran (Thermo Fisher Scientific, Waltham, MA). Approximately 1–2 pL (pL) was injected into each newly fertilized egg based on the size of the injection bolus at about one-fifth of the egg diameter. Tbr, Alx1 and Ets1/2 MASOs were designed based on sequence information available from the sea urchin genome (echinobase.org) and purchased from Gene Tools, LLC (Philomath, OR) (Ets MASO sequence: 5′ GAA​CAG​TGC​ATA​GAC​GCC​ATG​ATT​G 3’; Alx1 MASO sequence: 5′ TAT​TGA​GTT​AAG​TCT​CGG​CAC​GAC​A 3′; Tbr MASO sequence: 5′ TGT​AAT​TCT​TCT​CCC​ATC​ATG​TCT​C 3′).

A genetic construct, *ΔLv-Cadherin* (gift from D. McClay, Duke University), which contains the truncated cytoplasmic tail of cadherin that sequesters β-catenin of the cWnt pathway, was used at 300 ng/μl as previously described to abolish its function as a transcriptional co-activator in cWnt-responsive cells (Miller and McClay, 1997; Logan et al., 1999). Animalized embryos were observed with the *ΔLv-Cadherin* injection (Supplementary Figure S1A).

We injected 2mM of Alx1 MASO, 0.7 mM Tbr MASO, and 2 mM Ets1 MASO based on prior studies (Oliveri et al., 2002; Ettensohn et al., 2003; Rafiq et al., 2014). For all MASOs, we observed expected phenotypes as previously described: Alx1 and Ets1 MASO resulted in no PMCs or skeleton (Ettensohn et al., 2003; Rafiq et al., 2014) and Tbr MASO resulted in complete loss of skeleton (Oliveri et al., 2002) (Supplementary Figure S1B).

All embryos were collected at the mesenchyme blastula stage at 24 hpf.

### 2.4 microRNA qPCR

500-1000 embryos from control or drug treatment were collected to examine the levels of miRNAs. For injected embryos, 200 embryos of control-injected and MASO-injected embryos were collected at mesenchyme blastula stage (24 h post fertilization; hpf). Purification and isolation of miRNAs were conducted using miRNeasy Mini Kit from Qiagen (Germantown, MD, Cat# 217004). cDNA synthesis from 100 ng total RNA was performed with miRCURY LNA RT Kit (10 µL volume reaction) which adds a 5’ universal tag of a poly(A) tail to mature miRNA templates (QIAGEN, Germantown, MD). cDNA template was diluted 1:10, and miRNA qPCR was performed using miRCURY LNA miRNA PCR Assays (QIAGEN, Germantown, MD) in QuantStudio 6 Real-Time PCR cycler system (Thermo Fisher Scientific, Waltham, MA). Sea urchin miR-200 was used as a normalization controls due to its similar expression from cleavage to larval stages (Song et al., 2012; Konrad et al., 2023). Results are shown as fold changes comparing the control and the experimentally-treated (drug or MASO/construct-injected) mesenchyme blastula embryos using the Ct^-2ΔΔ^ method (Livak and Schmittgen, 2001; Stepicheva et al., 2015; Konrad et al., 2023). Custom miRCURY LNA miRNA PCR Primer Mix against various miRNAs were purchased from QIAGEN (Supplementary Table S2). miRNA seed sites are underlined.

All embryos were collected at the mesenchyme blastula stage (24 hpf). This particular time point of mesenchyme blastula stage was chosen because we wanted to capture miRNA dynamics at a time when these miRNAs are expressed and that when various cell types from the three germ layers are in the process of becoming specified. For data analyses, we identified outliers by finding data points outside the third quartile range via box-and-whisker plot (Burns et al., 2005). Outliers were removed from the final analysis. We then analyzed statistical significance with the 2-tailed heteroscedastic Student’s t-test.

### 2.5 Computational prediction of TF binding sites regulating miRNAs

To identify potential TF binding sites within select miRNA genes, we used the algorithm FIMO, a part of the MEME-Suite, to identify individual putative TF binding sites (Grant et al., 2011; Bailey et al., 2015). The search method used by FIMO uses position weighted matrices for motif data, which accounts for the inherent variability of functional TF binding sites found throughout genomes (Muppirala et al., 2011). A position weighted matrix (PWM) is a matrix containing information on the probability of a given nucleotide occurring at a given position within a motif. FIMO scans for instances of these short nucleotide motifs located within a larger nucleotide sequence. Both the genomic regions in which TFs are expected to bind as well as binding motifs for individual TFs were acquired from Echinobase, a database containing genomic information for *S. purpuratus* and other echinoderms. We also obtained information from CIS-BP (Catalogue of Inferred Sequence-Binding Preferences), a database containing binding motif information for DNA-binding proteins, along with the MEME-Suite database containing the CIS-BP motifs in MEME format (Weirauch et al., 2014; Arshinoff et al., 2022; Telmer et al., 2024). Genomic sequences for regions upstream of screened miRNAs were acquired from Echinobase using the Reference Sequence track (Grant et al., 2011). We pulled a region encompassing the first 10 kb upstream of each miRNA locus, based on literature suggesting that binding sites for TFs tend to fall within the proximal region upstream of the transcription start site (Lin et al., 2010; Whitfield et al., 2012). For this investigation, we define the beginning of the search region as either the first base of the annotated miRNA feature in Echinobase, if the miRNA is annotated, or, if it is not, the beginning of the BLASTn alignment of the miRNA precursor sequence (as catalogued in miRbase) to the *S. purpuratus* genome. To evaluate which experimental results were statistically significant, and thus warranted analysis of bioinformatic predictions, fold changes in miRNA levels determined from qPCR data using the Ct^-2ΔΔ^ method were compared to the control miR-200 using a two-tailed Student’s t-test (Livak and Schmittgen, 2001; Konrad and Song, 2023). Following generation of a list of predicted binding sites on the same strand of DNA as the miRNA locus, the statistical significance of each predicted site was used to evaluate predictions, with a threshold of *p*<(1 × 10^−5^) applied to filter algorithmic output. Individual predicted binding sites were additionally evaluated based on whether their genomic coordinates had ATAC-seq reads in the genome. ATAC-seq data is gathered from chromatin exposed to Tn5 transposases, which bind and cleave accessible DNA (Buenrostro et al., 2013; 2015; Shashikant et al., 2018). This cleaved DNA is then sequenced, yielding an alignment to the genome wherever DNA is not closed off by chromatin, and thus open to be bound by TFs and transcribed.

## 3 Results

### 3.1 Wnt signaling perturbation leads to miRNA transcript changes

**FIGURE 1:**
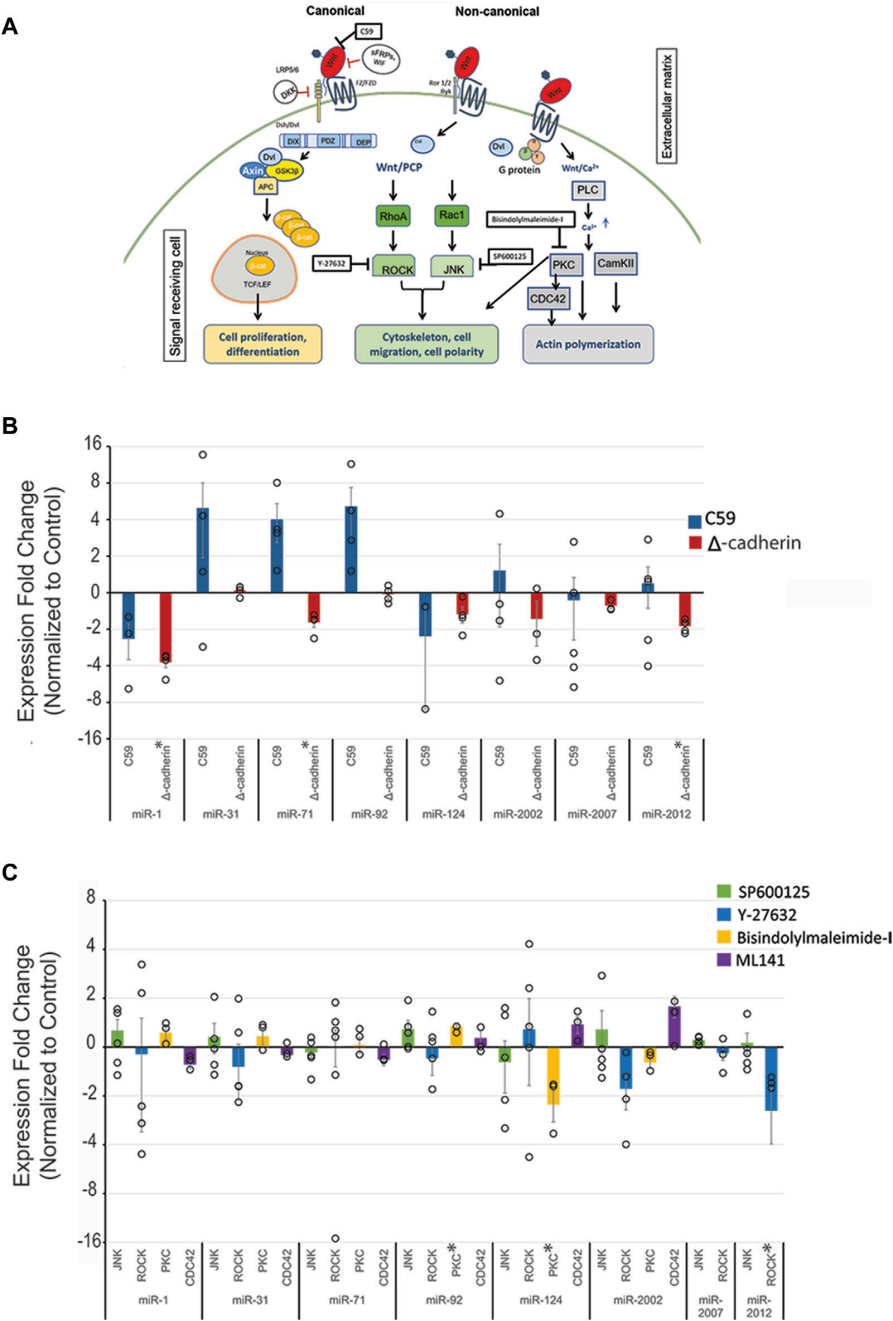
Wnt signaling regulates *Sp*miR-1, *Sp*miR-71, *Sp*miR-92, *Sp*miR-124, and *Sp*miR-2012 levels. **(A)** Schematic of Wnt signaling pathways. Modified from Song et al., 2015. **(B)** Sea urchin zygotes were treated with C59 which abrogates all branches of Wnt pathways. Zygotes were injected with truncated cadherin to perturb the canonical Wnt signaling pathway. This was followed by qPCR against various miRNAs, including evolutionarily conserved miRNAs (miR-1, miR-31, miR-92, miR-124), as well as some echinoderm-specific miRs (miR-2007, miR-2002, miR-2012). qPCR data indicate effects on miRNA expression following perturbation of canonical Wnt signaling. Each replicate is indicated by the circle. Three to five replicates were conducted. **(C)** qPCR data showing effects on miRNA expression following perturbation of non-canonical Wnt signaling pathways. Results indicate that Wnt perturbation results in variable levels of miRNAs. *p < 0.05 for 2-tailed heteroscedastic Student’s t-test. Each replicate is indicated by the circle. Three to five replicates were conducted. Standard Error of the Mean (SEM) is graphed.

The function of Wnt signaling is highly evolutionarily conserved, where perturbation results in similar anterior (head) to posterior (tail) axis defects or gastrulation defects in diverse metazoan species (Goldstein et al., 2006; Dunty et al., 2008; Gurley et al., 2008; Petersen and Reddien, 2009). To examine if the Wnt signaling pathway regulates select miRNAs, we used several inhibitors against the Wnt pathways. C59 inhibits the activity of porcupine, which is required for Wnt ligand palmitoylation, secretion, and biological function (Proffitt et al., 2013; Cui et al., 2014; Motono et al., 2016) (Figure 1A). Thus, Wnt-C59 inhibitor was used to inhibit both cWnt and ncWnt signaling pathways. Perturbation of all Wnt signaling with C59 did not result in significant changes in levels for any miRNAs (Figure 1B).

To further dissect the effect of individual branches of Wnt signaling on the expression of this select set of miRNAs, cWnt and ncWnt signaling pathways were inhibited separately. To inhibit cWnt signaling, we microinjected a genetic construct, *ΔLv-Cadherin*, which contains the truncated cytoplasmic tail of cadherin that sequesters β-catenin of the cWnt pathway, abolishing its function as a transcriptional co-activator in cWnt-responsive cells (Miller and McClay, 1997; Logan et al., 1999). We demonstrated that *ΔLv-Cadherin* injection resulted in expected phenotype of embryos lacking the endomesoderm (Supplementary Figure S1A). Using this approach to block the cWnt/β-catenin signaling, we observed significant decreases in the levels of *Sp*miR-1, *Sp*miR-71, and *Sp*miR-2012, suggesting that the cWnt/β-catenin pathway may transcriptionally activate or stabilize these miRNAs (Figure 1B).

We used Y-27632 (Rangel-Mata et al., 2007), a small molecule inhibitor against ROCK. ROCK is an effector of ncWnt/PCP signaling, and is also activated by VEGF signaling to regulate gene expression in sea urchin skeletogenic cells that impact spicule formation and biomineralization (Rangel-Mata et al., 2007; Hijaze et al., 2024). Results indicate that perturbation of ncWnt/PCP with ROCK inhibitor leads to a significant decrease in *Sp*miR-2012 level (Figure 1C). Inhibition of ncWnt/PCP with SP600125, which is a selective, reversible and ATP-competitive inhibitor of JNK, had no significant impact on the set of miRNAs tested (Figure 1C) (Bennett et al., 2001). Downstream of ncWnt/PCP, Cdc42 is one of the effector proteins activated to modulate cell polarization and migration (Alford et al., 2009; Surviladze et al., 2010; Moorhouse et al., 2015; Sepúlveda-Ramírez et al., 2018). Inhibition of Cdc42 with ML141 also did not result in any significant changes in miRNA levels (Figure 1C) (Surviladze et al., 2010). Downstream of the ncWnt/Ca^2+^, we used Bisindolylmaleimide-I to inhibit PKC (Toullec et al., 1991; Gekeler et al., 1996). We observed that there was a small but significant increase of *Sp*miR-92 and a significant decrease of *Sp*miR-124.

### 3.2 Nodal signaling regulates *Sp*miR-31 and *Sp*miR-2012

**FIGURE 2:**
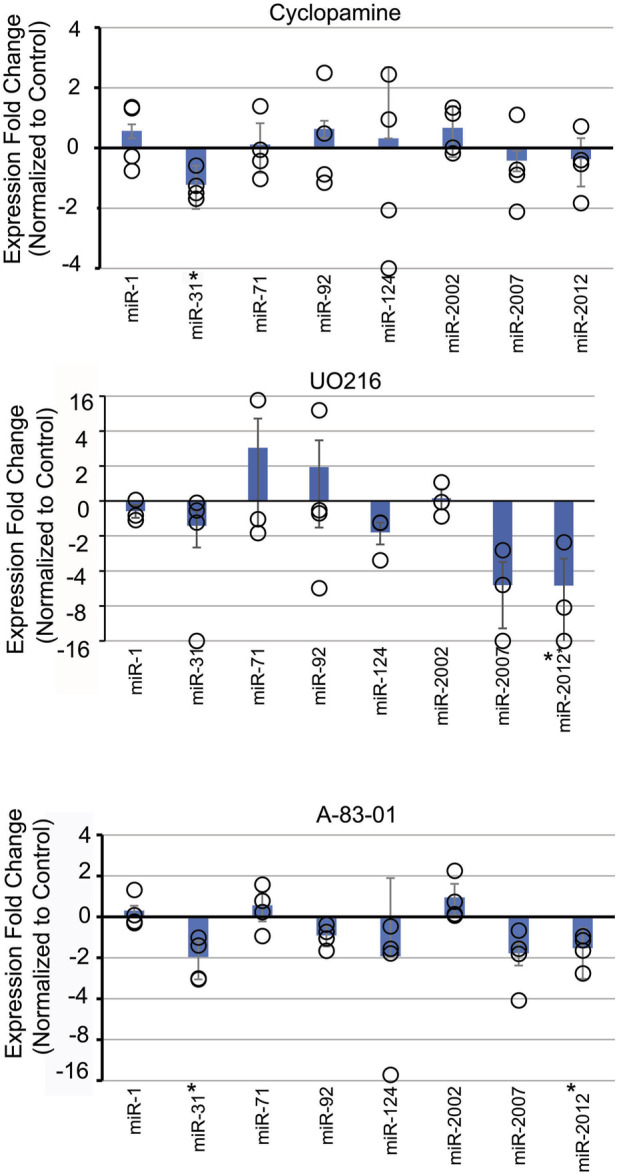
Inhibition of various signaling pathways results in selective effects on *Sp*miRNA levels. Sea urchin zygotes were incubated with inhibitors of various signaling pathways. Cyclopamine was used to inhibit Sonic Hedgehog (SHH). UO216 was used to inhibit the MAPK pathway. A-83-01 was used to inhibit Nodal signaling pathway. This was followed by qPCR against miRNAs. *p < 0.05 for 2-tailed heteroscedastic Student’s t-test. Three replicates were conducted. Standard Error of the Mean (SEM) is graphed.

To test the impact of Nodal signaling on the level of miRNAs, we used inhibitor SB431542, which specifically inhibits Alk4/5/7 receptors of the Nodal/Activin pathway by acting as a competitive ATP binding site kinase inhibitor (Inman et al., 2002; Tojo et al., 2005). Another Nodal inhibitor, A-83-01, with lower IC_50_ was also used (Tojo et al., 2005). Nodal perturbation with SB431542 did not result in any significant changes for the miRNAs tested (Supplementary Figure S2). However, perturbation of Nodal signaling with A-83-01 resulted in significant decrease in *Sp*miR-31 and *Sp*miR-2012 levels (Figure 2). This difference in results could be due to the lower IC_50_ of A-83-01.

### 3.3 Disruption of MAPK signaling decreases level of *Sp*miR-2012

Treatment with U0126, a kinase inhibitor which selectively inhibits MEK1 and MEK2 activation, results in inhibition of MAPK/ERK (Rottinger et al., 2004). In the sea urchin, MAPK/ERK has been shown to be involved in development of the micromere lineage and skeletogenesis in sea urchin embryos (Rottinger et al., 2004). Treatment of embryos with U0126 resulted in a significant decrease of *Sp*miR-2012 (Figure 2).

### 3.4 Perturbation of Sonic Hedgehog signaling pathway results in decreased miR-31 levels

Cyclopamine is a small molecule teratogenic alkaloid which directly interacts with and inhibits Smoothened, a G-protein coupled receptor critical for SHH signaling (Batsaikhan et al., 2014). The SHH pathway is involved in patterning of the mesoderm and L/R axis in sea urchin embryos (Walton et al., 2009; Warner et al., 2016). Treatment with Cyclopamine resulted in a small but significant decrease of *Sp*miR-31 level.

### 3.5 Perturbation of Delta/Notch, VEGF, and BMP signaling pathways did not result in significant changes in miRNA levels

We also tested the impact of additional key signaling pathways that are critical for development, including Delta/Notch, VEGF, and BMP signaling pathways (Supplementary Figure S2). We used pharmaceutical inhibitors, DAPT and Omeprazole, to block the Delta/Notch signaling pathway. DAPT inhibits γ-secretase, preventing downstream Delta/Notch signaling (Feng et al., 2019).

Omeprazole is a proton pump inhibitor that blocks H+/K + ATPase (Fellenius et al., 1981). Delta/Notch signaling has been shown to be dependent on H+/K + -ATPase for activation during L/R axis patterning in vertebrates and sea urchins (Raya et al., 2004; Bessodes et al., 2012). Omeprazole has additionally been shown to induce oligomerization of the Notch3 N-terminal fragment, resulting in destabilization of the protein (Young et al., 2021; 2022). We did not observe significant changes in the miRNA levels in response to DAPT or Omeprazole (Supplementary Figure S2).

To disrupt VEGF signaling, we used Axitinib, which acts as a selective inhibitor of VEGF RTK1/2/3 (Hu-Lowe et al., 2008; Adomako-Ankomah and Ettensohn, 2013). Inhibition of VEGF signaling did not result in any significant changes in miRNA levels (Supplementary Figure S2).

We also disrupted the BMP signaling pathway with Dorsomorphin, a drug which inhibits type I BMP receptors ALK2/3/6 (Hao et al., 2008; Yu et al., 2008). miRNA levels were not significantly altered upon disruption of BMP signaling (Supplementary Figure S2).

### 3.6 *Alx1*, *Ets1/2* and *Tbr* perturbation results in significant changes of select miRNAs

**FIGURE 3:**
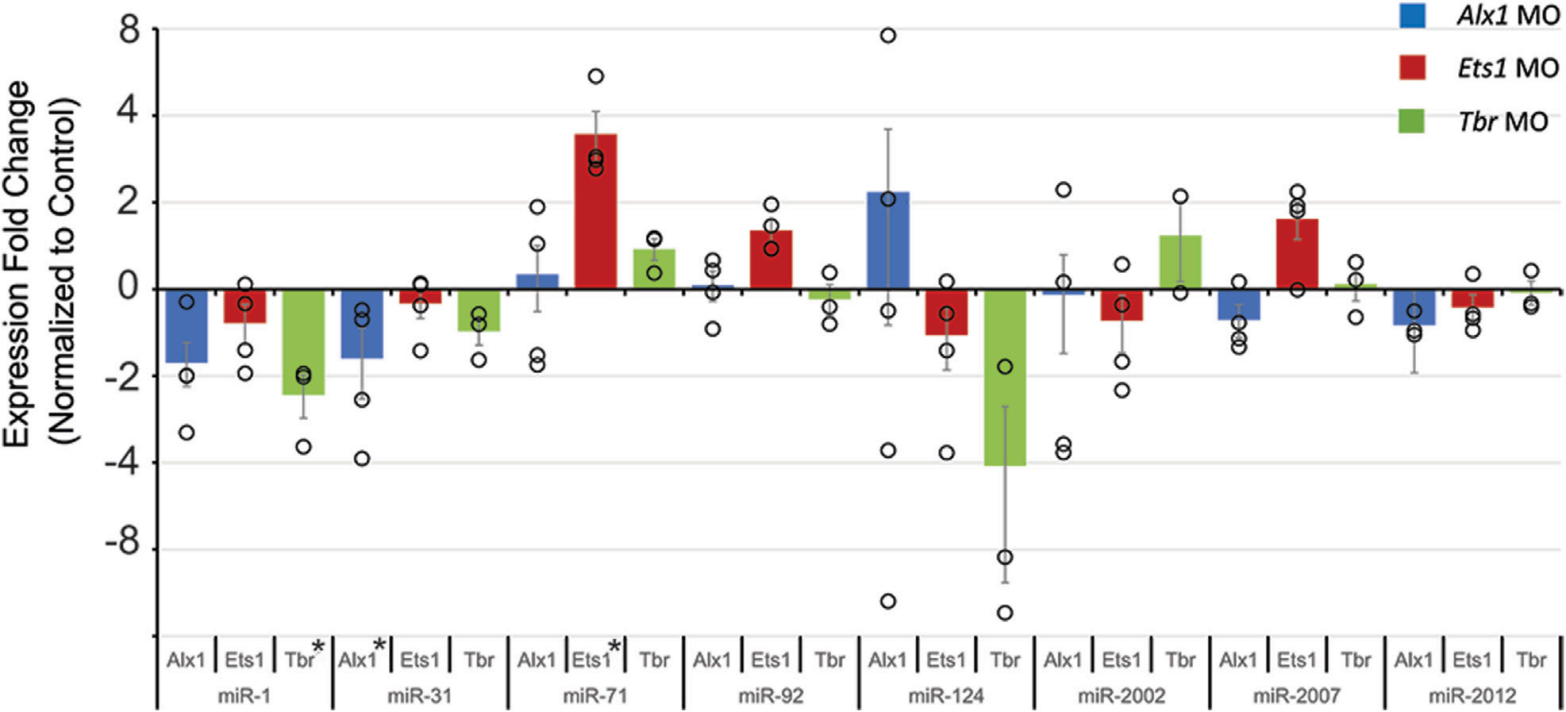
Developmental transcription factors Alx1, Ets1/2, and Tbrain regulate levels of *Sp*miR-1, *Sp*miR-124, and *Sp*miR-71. Sea urchin zygotes were injected with morpholino antisense oligonucleotides (MASOs) complementary to transcription factors Alx1, Ets1, and Tbr to prevent their translation. Results indicate that perturbation results in variable levels of miRNAs. *p < 0.05 for 2-tailed heteroscedastic Student’s t-test. Each replicate is indicated by the circle. Five replicates were conducted. Standard Error of the Mean (SEM) is graphed.

The GRN regulating PMC specification and skeletogenesis is well characterized in the sea urchin, with Alx1, Ets1/2, and Tbr known to be key TFs of the skeletogenic GRN (Kenny et al., 1999; Kurokawa et al., 1999; Croce et al., 2001; Fuchikami et al., 2002; Oliveri et al., 2002; 2008; Ettensohn et al., 2003; Howard-Ashby et al., 2006; Rizzo et al., 2006; Revilla-i-Domingo et al., 2007; Khor et al., 2019). Knockdown of Alx1, Ets1/2, and Tbr yielded predicted phenotypes of PMC and skeletal loss (Supplementary Figure S1B). To test if Alx1, Ets1/2 and Tbr regulate these selected *Sp*miRNAs, we examined *Sp*miRNA levels in embryos injected with loss-of-function reagents against these TFs. Results indicate that knockdown of *Alx1* resulted in a statistically significant decrease in *Sp*miR-31 levels; knockdown of *Tbr* resulted in a statistically significant decrease in *Sp*miR-1 levels; and knockdown of *Ets1/2* resulted in significant increase of *Sp*miR-71 levels (Figure 3).

### 3.7 Conservation of genomic structures used to identify conserved regulatory elements

The rationale for analyzing the conservation of genomic features in regions surrounding miRNAs is that genomic structures with protein coding sequence and *cis*-regulatory elements may be conserved among closely related organisms (Babarinde and Saitou, 2016). This is one of the criteria we set to identify potential *cis*-regulatory elements. Overall, we observed conservation of genomic features in the regions surrounding miRNAs between *S. purpuratus* (*Sp*) and the green sea urchin *Lytechinus variegatus* (*Lv*) (Supplementary Figure S3) (Arshinoff et al., 2022; Telmer et al., 2024). However, we noted the genomic context surrounding the *Sp*miR-1 genomic loci in a few closely related echinoderm species is different, where the region surrounding miR-1 was annotated as an intron of *Mib1* in *L. variegatus* and *A. planci*, but annotated as intergenic region in *P. miniata* and *S. purpuratus* (Supplementary Figure S4). This distinction is important to resolve, as co-transcription of the intronic miRNA with the host gene may be a relevant mechanism of transcriptional control, in addition to independent transcriptional regulation of the miRNA. The coding regions on either side of miR-1 are similar across all species, and the miR-1 locus in *S. purpuratus* is an intergenic region between two genes both annotated as *Mib1* (Supplementary Figures S3, S4). We used distantly related human and mouse genomes to compare the miR-1 locus and found that miR-1 in these mammalian species suggested an intronic *Sp*miR-1 (Supplementary Figure S4). To test this possibility, we examined the genomic locus of *Sp*miR-1, by designing several PCR primer pairs to amplify *Mib1* and *Sp*miR-1 regions (Supplementary Figure S4). If miR-1 was intergenic, we would not expect a PCR product, since it would be over 100 kilo base pairs. The control primers within *Mib1* exons on either side of miR-1 produced the expected PCR bands of the correct sizes. Using primers spanning *Mib1* exons on either side of miR-1, we observed a PCR product of the expected size, consistent with an intronic *Sp*miR-1. Sequencing results indicate that the PCR products align within the *Mib1* exons. Thus, these results indicate that *Sp*miR-1 is likely to be intronic (Supplementary Figure S4), suggesting a striking preservation of the genomic environment surrounding miR-1 that extends from the sea urchin all the way into mouse and human genomes (Rangwala et al., 2021) (Supplementary Figure S4).

### 3.8 Bioinformatic analysis to identify potential transcription factors that directly regulate miRNAs

With genomic sequences taken from Echinobase and short nucleotide motifs (represented as PWMs) taken from CIS-BP, FIMO scans were used to identify individual putative TF binding sites. For each identified putative TF binding site, the FIMO search results include the coordinates and sequence of the site within the provided genomic region, as well as *p*-value and alignment score, the associated CIS-BP motif, and an ID for the individual predicted transcription factor. Once predicted binding sites associated with individual genes were identified, we conducted a literature search to identify developmental signaling pathways associated with each gene. The resulting list of predicted TFs associated with specific signaling pathways regulating each miRNA was compared against experimental data on miRNA regulation by various signaling pathways. The described searches and analyses were performed in both *S. purpuratus* and *L. variegatus* genomes to provide additional evidence of evolutionary conservation of predicted binding sites. One further line of evidence that was employed was ATAC-seq data made available in Echinobase (Arshinoff et al., 2022; Telmer et al., 2024). ATAC-seq data indicate loci in the genome which are maintained as open euchromatin, available to TF binding and transcription (Buenrostro et al., 2013; 2015; Shashikant et al., 2018; Shashikant and Ettensohn, 2019). Thus, we use the ATAC-Seq data as an additional way to assess potential TF binding during development.

**FIGURE 4:**
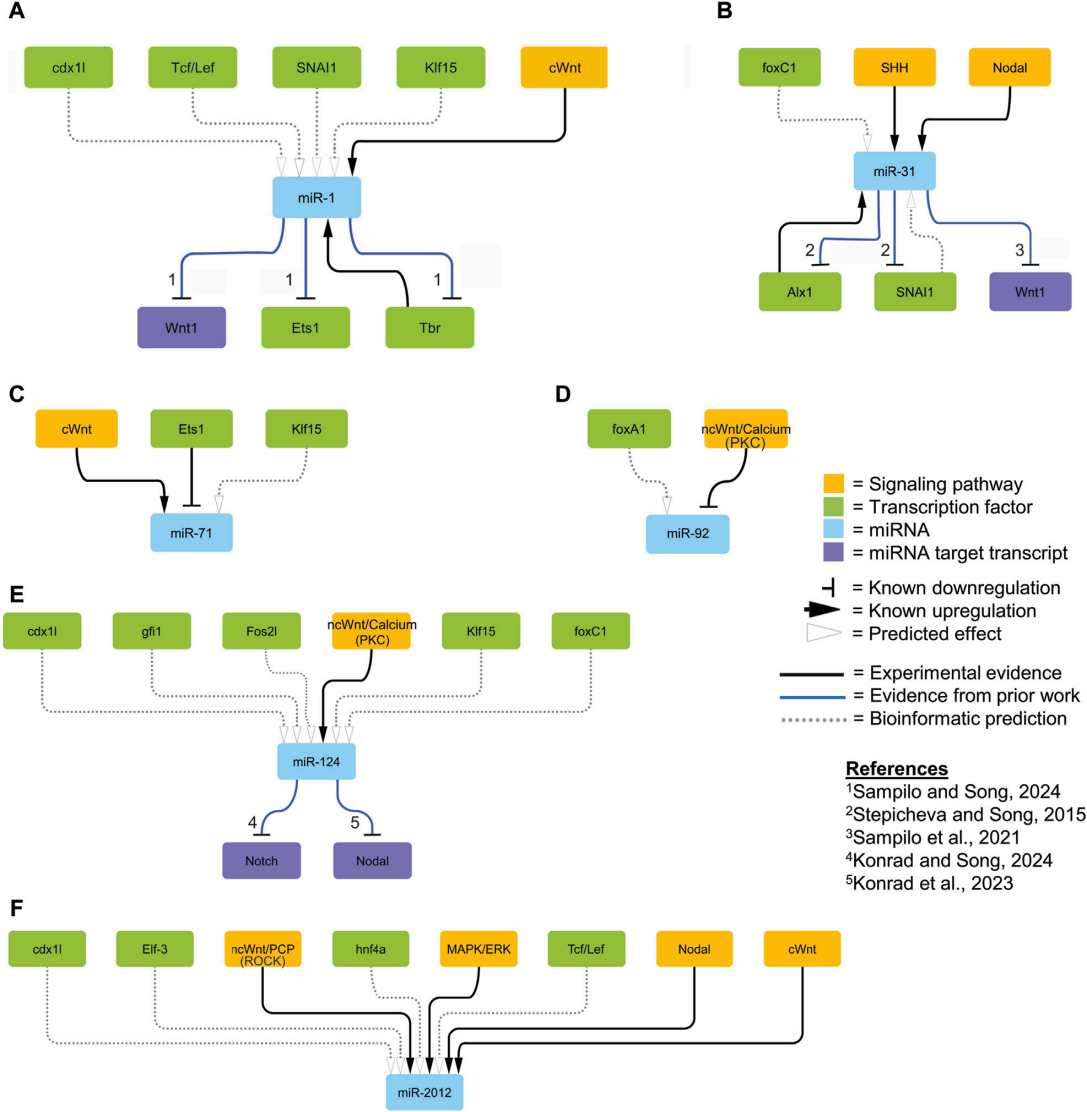
Proposed regulatory network. We integrated relevant regulatory interactions identified in experimental results (solid black lines; Figures 1–3), those predicted by bioinformatics (dashed lines; Table 1), and those described in the literature (solid blue lines) within the sea urchin embryo into a network visualized using Cytoscape (ver 3.10.1) (Shannon et al., 2003). The orange colored nodes represent components of the signaling pathway; green colored nodes represent transcription factors; blue colored genes represent miRNAs; and purple nodes represent specific miRNA target transcripts. The horizontal bars indicate known downregulation; the black arrows indicate known upregulation; the open arrows indicate predicted effects based on bioinformatics analyses. The numbers correspond to specific references.

Results indicate that *Sp*miR-1 levels are affected by cWnt signaling and Tbr (Figure 1B, Figure 3). For the region upstream of *Sp*miR-1, FIMO predicted binding sites for Kruppel-like factor 15 (Klf15) and SNAI1 (Table 1; Figure 4A), both of which are regulated by Wnt signaling (Horvay et al., 2011; Noack et al., 2019). Among the total list of TF binding sites identified using FIMO, these TFs were associated with Wnt and ꞵ-catenin signaling, with motifs located within 100-150bp of the start of annotated *Sp*miR-1 in Echinobase. The *Sp*miRNA sequence annotations in Echinobase are ∼100bp on average, with BLAST alignments of known *Sp*miRNA sequences showing the annotations extending beyond the alignment of the precursor sequence, suggesting that the locus annotation encompasses the stem-loop forming portion of the *Sp*miRNA transcripts. ATAC-seq reads were found overlapping the predicted binding sites for Klf15 and SNAI1 upstream of this *Sp*miR-1 locus at multiple timepoints between 24 and 60 hpf, indicating that the sites are maintained as open euchromatin during early development (Buenrostro et al., 2013; Buenrostro et al., 2015; Shashikant and Ettensohn, 2019; Arshinoff et al., 2022; Telmer et al., 2024). Further upstream, we identified potential binding sites for HMG protein Tcf/Lef (566bp upstream), and caudal type homeobox 1-like, all of which are also regulated by Wnt signaling (Tcf/Lef and caudal are upregulated by cWnt) (Novak and Dedhar, 1999; Lickert et al., 2000; Noack et al., 2019). *Lv*miR-1 (within 150 bp) did not yield any predicted binding sites for the same factors identified in the purple sea urchin at similar distances from *Sp*miR-1 (Table 1, Supplementary Table S3). A binding site for SNAI1 was identified (1,203bp) upstream of *Lv*miR-1. A bioinformatic screen for TF binding sites indicated a binding site for Tbr at 9,488bp upstream of the *Sp*miR-1 locus (Table 1). This particular prediction is corroborated in *Lv*miR-1, where a Tbr binding site is predicted at 6,256bp (Table 1).

**TABLE 1:** Processed FIMO Results for miRNAs in *Strongylocentrotus purpuratus* (Sp) and *L. variegatus* (Lv).

miRNA	Gene name	Motif type	Regulated by	Up vs Downregulation	*p*-value	q-value^a^	Distance from miRNA	Do hits overlap with ATAC-seq?	References
miR-1	Kruppel like factor 15	C2H2 ZF	Wnt	Down	SP:8.83E-05	SP:0.0743	SP:102	SP: 24, 30, 50, 60 h	Noack et al. (2019)
					LV:7.37E-05	LV:0.163	LV:963	LV: EC, MC, LC, HB, MG, EL	
	snail family transcriptional repressor 1 (SNAI1)	C2H2 ZF	Wnt, SHH	Up	SP:3.79E-05	SP:0.748	SP:140	SP: 30, 60 h	Horvay et al. (2011), Heiden et al. (2014)
					LV:3.74E-05	LV:0.747	LV:1203	LV: EC, MC, LC, HB, MG, EL	
	HMG protein Tcf/Lef	Sox	Wnt	Up	SP:3.66E-05	SP:0.355	SP:566	SP: 18, 24, 39 h	Novak and Dedhar (1999)
					LV:8.22E-05	LV:0.227	LV:963	LV: MC, LC, HB, MG, EL	
	caudal type homeobox 1-like	Homeodomain	Wnt/β-catenin	Up	SP:5.22E-06	SP:0.094	SP:8085	SP:39 h	Lickert et al. (2000)
					LV:7.12E-05	LV:0.419	LV:3089	LV: EC, MC, LC, HB, MG, EL	
	T-box brain transcription factor 1 (Tbr)	T-box_direct_M00733_2.00	Regulates miR-1	Up	SP: 8.94E-05	SP:1	SP:9488	SP: 18, 24, 39, 60 h	
					LV: 2.78E-05	LV:0.184	LV: 6256	LV: MC, LC, HB, MG, EL	
miR-31	snail family transcriptional repressor 1 (SNAI1)	C2H2 ZF	Wnt, SHH	Up	SP:3.96E-05	SP:0.541	SP:469	SP: 18, 24, 30, 39, 50, 60 h	Horvay et al. (2011), Heiden et al. (2014)
					LV:4.78E-07	LV:0.00955	LV:275	LV: NONE	
	forkhead box C1	Forkhead	TGF-β	Up	SP:7.50E-05	SP:0.525	SP:4051	SP:NONE	Massagué (1998), Zhang et al. (2019)
					LV:6.47E-05	LV:0.193	LV:3847	LV: EC, MC, LC, HB, EL	
miR-71	Kruppel like factor 15	C2H2_ZF_inferred_M08323_2.00	Wnt	Down	SP:8.46E-07	SP:0.00564	SP:7127	SP: NONE	Noack et al. (2019)
					LV:7.10E-05	LV:0.0881	LV:4524	LV: ALL	
	Ets1	Ets	Regulates miR-71	Down	SP: 3.31E-05	SP:0.66	SP:32	SP: 18, 60, 70 h	
					LV: 2.99E-05	LV:0.594	LV:2105	LV: MC, LC, HB, MG	
miR-92	forkhead box A1	Forkhead	PKC	Up	SP:9.50E-05	SP:0.573	SP:1064, 3238	SP: NONE	Johnson et al. (2012)
					LV:6.09E-05	LV:0.369	LV:7268, 7728	LV: EC, MC, LC, HB, MG, EL	
miR-124	caudal type homeobox 1-like	Homeodomain	cWnt	Up	SP:3.27E-05	SP:0.551	SP:4887	SP:NONE	Lickert et al. (2000)
					LV:5.73E-05	LV:0.227	LV:380	LV: EC, LC, HB, MG, EL	
	forkhead box C1	Forkhead_inferred_M00257_2.00	TGF-β	Up	SP:2.35E-05	SP:0.232	SP:8945	SP: 30, 39 h	Massagué (1998), Zhang et al. (2019)
					LV:9.58E-05	LV:0.215	LV:4411	LV: EC, LC	
	Fos-related antigen 2-like	bZIP	Delta/Notch/IL1β	Down	SP:8.19E-06	SP:0.135	SP:1689	SP: 18, 24, 30, 39, 50, 60, 70 h	Choi et al. (2021)
					LV:3.40E-07	LV:0.00583	LV:1284	LV: MC, LC, HB, MG	
	growth factor independent 1 transcriptional repressor	C2H2 ZF	Delta/Notch	Down	SP:9.79E-05	SP:1	SP:10037	SP:18, 30, 50	Franco et al. (2006)
					LV:6.37E-05	LV:0.384	LV:474	LV: MC, LC, HB, MG	
	Kruppel like factor 15	C2H2_ZF_inferred_M08323_2.00	Wnt	Down	SP:7.69E-05	SP: 0.0893	SP:2069	SP: 18, 24, 30, 39, 60, 70	Noack et al. (2019)
					LV:3.69E-06	LV:0.0741	LV:1821	LV: MC, LC, HB, EL	
miR-2012	caudal type homeobox 1-like	Homeodomain	cWnt	Up	SP:4.63E-05	SP:0.542	SP:5499	SP: 18, 24, 30, 39, 50, 60, 70 h	Lickert et al. (2000)
					LV:7.73E-05	LV:0.27	LV:4722	LV: MC, LC, HB, EL	
	ETS-related transcription factor Elf-3	Ets_inferred_M07944_2.00	MAPK	Up	SP:6.35E-05	SP:0.21	SP:719	SP: 18, 39, 50 h	Chen et al. (2018)
					LV:1.91E-05	LV:0.188	LV:1710	LV: MC, LC, HB, MG, EL	
	hepatocyte nuclear factor 4 alpha	Nuclear_receptor_inferred_M08219_2.00	cWnt	Down	SP:5.27E-07	SP:0.0105	SP:5133	SP: 18, 24, 30, 39, 50, 60, 70 h	Yang et al. (2013)
					LV:5.99E-05	LV:0.401	LV:1089	LV: MC, LC, HB, MG, EL	
	HMG protein Tcf/Lef	SOX	Wnt	Up	SP:7.02e-07	SP:0.0135	SP:6518	SP: 18, 30, 39, 50, 60, 70 h	Novak and Dedhar (1999)
					LV:5.10e-05	LV:0.355	LV:653	LV: MC, LC, HB, MG, EL	

^a^ The q-value measures false positive rate (Storey and Tibshirani, 2003).

We found *Sp*miR-31 levels to be affected by Alx1, SHH, and Nodal signaling (Figures 1–3). No sequences matching the Alx1 binding motif were located upstream of *Sp*miR-31 (Shashikant and Ettensohn, 2019; Arshinoff et al., 2022; Telmer et al., 2024). A predicted binding site for SNAI1, a TF shown to be upregulated by cWnt and SHH signaling (Horvay et al., 2011; Heiden et al., 2014), was found 469 bp upstream of the *Sp*miR-31 locus, with the binding site having ATAC-seq reads throughout early development (Arshinoff et al., 2022; Telmer et al., 2024) (Table 1; Figure 4B). We bioinformatically identified a binding site for Forkhead Box C1 (upregulated by TGF-β signaling) 4,051bp upstream of *Sp*miR-31 (Massagué, 1998; Zhang et al., 2019) (Table 1). However, this locus did not overlap with any ATAC-seq reads for any timepoint in early development of *S. purpuratus* sea urchin, but does overlap with ATAC-seq for *L. variegatus* sea urchin. In *Lv*miR-31, binding sites for SNAI1 and Forkhead box C1 were identified at similar distances to the sites identified in *Sp*miR-31 (275 bp upstream for *Lv*SNAI1 and 3,847 for *Lv*Forkhead Box C1) (Table 1).

The level of *Sp*miR-71 was significantly affected by cWnt and Ets1/2. The level of *Sp*miR-71 was significantly decreased upon cWnt disruption with the *ΔLv-Cadherin* injection, suggesting that it may be positively regulated by cWnt signaling (Figure 1B; Figure 4C). We identified TFs regulated by cWnt that had predicted binding sites for Kruppel like factor 15 within 10kb upstream of *Sp*miR-71 (Table 1). This predicted binding site overlaps with ATAC-seq reads between 18 and 70 hpf (Arshinoff et al., 2022; Telmer et al., 2024). A bioinformatically predicted binding site for Ets1 was identified at 32 bp upstream of the *Sp*miR-71 locus (Table 1), consistent with Ets1/2 loss-of-function leading to increased *Sp*miR-71 level (Figure 3). In addition, a binding site for Ets1 was found, but farther upstream of *Lv*miR-71 (2,105bp) compared to *Sp*miR-71 (32 bp) (Table 1).

We found the level of miR-92 to be significantly increased upon Bisindolylmaleimide-I treatment against ncWnt/Ca^2+^ pathway (Figure 1C). Predicted binding sites for Forkhead box A1 (regulated by PKC) were found 1064bp and 3238bp upstream of *Sp*miR-92 (Table 1) (Johnson et al., 2012). This prediction was corroborated in *Lv*miR-92, with predicted sites at 7268 bp and 7728 bp upstream of *Lv*miR-92 (Table 1; Figure 4D).

We found the level of *Sp*miR-124 to be significantly decreased upon disruption of the ncWnt/Ca^2+^ signaling pathway (Figure 1C). However, we did not find predicted binding sites for TFs regulated by PKC upstream of *Sp*miR-124 or *Lv*miR-124 (Table 1; Figure 4E).

The level of *Sp*miR-2012 was found to be significantly decreased by disruption of cWnt, ncWnt/PCP (ROCK), Nodal, and MAPK signaling pathways (Figure 1B; Figure 2). Predicted binding sites for Wnt-regulated TFs were identified, including caudal type homeobox 1-like (upregulated by cWnt), hepatocyte nuclear factor 4 alpha (downregulated by cWnt), and HMG protein Tcf/Lef (downregulated by cWnt) (Table 1; Figure 4F) (Novak and Dedhar, 1999; Lickert et al., 2000; Yang et al., 2013; Noack et al., 2019). Sites for caudal type homeobox 1-like, hepatocyte nuclear factor 4 alpha, and HMG protein Tcf/Lef overlapped with ATAC-seq reads between 18 and 70 hpf. Of the predicted binding sites for TFs found upstream of *Sp*miR-2012, none were for TFs regulated by Nodal signaling (Table 1). A predicted binding site for Elf-3, a TF upregulated by MAPK signaling (Chen et al., 2018), was identified at 719bp upstream of the *Sp*miR-2012 locus, coinciding with ATAC-seq reads present during the larval stage (Arshinoff et al., 2022; Telmer et al., 2024). In *Lv*miR-2012, TFs downstream of cWnt signaling, including caudal-type homeobox 1-like hepatocyte nuclear factor 4 alpha, and HMG protein were predicted to bind upstream of *Lv*miR-2012, corroborating predictions for *Sp*miR-2012 (Table 1).

## 4 Discussion

We identified signaling pathways and transcription factors active during embryogenesis which may potentially regulate the transcript levels of several miRNAs. Signaling pathways were found to regulate *Sp*miR-1, *Sp*miR-31, *Sp*miR-71, *Sp*miR-92, *Sp*miR-124, and *Sp*miR-2012 (Figures 1, 2). We also found Tbr, Alx1, and Ets1/2 to regulate *Sp*miR-1, *Sp*miR-31, and *Sp*miR-71, respectively (Figure 3). With our experimental data, we used bioinformatic predictions, evolutionary conservation, and existing ATAC-Seq information (Arshinoff et al., 2022; Telmer et al., 2024) to identify TFs which may mediate the transcript levels of these miRNAs (Figure 4). A notable implication of several results was the possibility of negative feedback loops governing the regulation of miRNAs and their targets.

Of note is that we did not find *Sp*miR-2002 and *Sp*miR-2007 to be responsive to any perturbations, indicating that these miRNAs are not regulated by these pathways and TFs and/or they may be functional later in development. Of the factors we have discovered to regulate *Sp*miR-1 levels, *Tbr* is previously characterized as a target of *Sp*miR-1 (Sampilo and Song, 2024). The echinoderm Tbr proteins are orthologous to vertebrate Eomesodermin (Eomes) (also known as Tbr2), Tbr1, and Tbx21 (also called T-bet) (Papaioannou and Silver, 1998; Croce et al., 2001). In the sea urchin, *Tbr* is zygotically expressed in the skeletogenic mesoderm of the cleavage and blastula stage embryo (Croce et al., 2001; Oliveri et al., 2002). Tbr is involved in skeletogenic mesoderm specification, as well as skeletogenesis in the larva, with *Tbr* loss-of-function resulting in complete loss of the larval skeleton (Croce et al., 2001; Fuchikami et al., 2002; Hinman et al., 2007; Gao and Davidson, 2008; Oliveri et al., 2008). We have previously found that *Sp*miR-1 overexpression results in mispatterning of the skeletogenic cells and duplicated branching of the larval skeleton (Sampilo and Song, 2024). We showed that *Sp*miR-1 inhibited blastulae have significantly increased *Tbr* mRNA levels compared to the control (Sampilo and Song, 2024). Here we found that *Tbr* knockdown results in a significant decrease in *Sp*miR-1 (Figure 3). It is interesting to note that expression of *Sp*miR-1 decreases in the early and mesenchyme blastulae and increases in the gastrula stage with enrichment in the mesenchymal cells (Sampilo and Song, 2024). Based on *Sp*miR-1 and *Tbr* expression data in the purple sea urchin, we speculate that while Tbr is not likely to be involved in inhibiting miR-1 expression in the blastula stage, it may be partly involved in activating miR-1 in the gastrula stage. Additionally, bioinformatic searches for *Tbr* binding sites identified one site at ∼9500 bp upstream of *Sp*miR-1, and 8,660 bp upstream in *Lv*miR-1 (Table 1). This set of data indicate that *Sp*miR-1 and Tbr are in a regulatory feedback loop where *Sp*miR-1 inhibits *Tbr* and Tbr activates *Sp*miR-1, suggesting that cross-regulation of miR-1 and Tbr may be important for proper skeletogenesis (Figure 4A).

Additionally, investigation of the *Sp*miR-1 genomic locus suggested that it was intronic, raising the possibility of co-transcription with its host gene, *Mib1* (Supplementary Figure S4). While it is possible for *Sp*miR-1 level to be entirely dependent on its host gene, previous research has shown that at least a third of miRNAs located within introns in *C. elegans* retained independent promoter regions, and as such potential mechanisms for independent regulation of miR-1 in the sea urchin should not be discounted (Isik et al., 2010). Existing literature on regulation of *Mib1* transcription is scarce, but does not suggest that it is regulated by Tbr or cWnt signaling (Kenny et al., 2001; Chen et al., 2021; 2023). This is inconsistent with our finding that *Sp*miR-1 level is significantly decreased upon Wnt perturbation with *ΔLv-Cadherin* injection, suggesting that inhibition of cWnt/β-catenin promotes the transcription or stabilization of miR-1 (Figure 1). Interestingly, Mib1 has been shown to activate cWnt signaling, suggesting a possible indirect mechanism of miR-1 regulation (Berndt et al., 2011).

In addition, previous work has shown miR-1 to inhibit components of the cWnt/β-catenin pathway. For example, miR-1 has been shown to inhibit *FZD7* in breast cancer cells and *Wnt1* ligand in the sea urchin (Liu et al., 2015; Sampilo and Song, 2024). In *Drosophila,* miR-1 directly suppresses *Prickle*, an essential ncWnt/PCP signaling component (King et al., 2011). Interestingly, miR-1 was found to suppress vertebrate oncogenic factor, *TCF7* of the cWnt/β-catenin pathway during prostate cancer (Siu et al., 2017). Thus, our prior work and other studies indicate that miR-1 regulates components of the Wnt signaling pathway, and the current work indicates that miR-1 itself is also regulated by the cWnt signaling pathway (Figures 1, 4A).

The C59 drug treatment did not result in significant changes of miRNA levels, whereas, injection of truncated cadherin leading to sequestering of β-catenin, resulted in significant changes in levels of *Sp*miR-1, *Sp*miR-71, and *Sp*miR-2012 (Figure 1B). The reason for this could be that injection of the truncated cadherin provides an immediate perturbation directly targeting the cWnt pathway. There may be maternal sources of Wnt ligands present that were not immediately affected by the C59 treatment in abrogating palmitoylation, secretion, and the biological activity of Wnt ligands. Treatment with Bisindolylmaleimide-I resulted in significant increase of *Sp*miR-92 and significant decrease of *Sp*miR-124 (Figure 1C). Also, treatment with ROCK inhibitor, Y-27632, resulted in significant decrease of *Sp*miR-2012. We do not understand why C59 treatment did not result in any significant changes compared to drugs against ncWnt/Ca^2+^ (Bisindolylmaleimide-I) and ncWnt/PCP (Y-27632).

Results indicate that loss-of-function of *Alx1* leads to significant decreases in the level of miR-31 (Figure 3). In addition, previous results have shown that miR-31 is a post-transcriptional regulator of *Alx1,* which is a primary driver of skeletogenic specification (Stepicheva and Song, 2015; Shashikant et al., 2018). Previously we have shown that miR-31 inhibition of specific block of miR-31’s suppression of *Alx1* leads to significant shortening of skeletal spicules and mispatterning of skeletogenic cells (Stepicheva and Song, 2015). *Sp*Alx1 mRNA is expressed specifically by skeletogenic cells throughout gastrulation (Ettensohn et al., 2003), and miR-31 is ubiquitously expressed in all cells throughout development (Stepicheva and Song, 2015). Knockdown of *SpAlx1* and *LvAlx1* revealed that Alx1 is essential for skeletogenic cell differentiation and skeletogenesis (Ettensohn et al., 2003). Potentially, Alx1 may be involved in activating *Sp*miR-31 to impact skeletogenesis, but the exact mechanism is not known. In vertebrates, Alx1 has known roles in neural crest development, and craniofacial structure specifically (Kayserili et al., 2009). Thus, prior and current work in the sea urchin indicate that miR-31 and Alx1 are in a possible feedback loop where Alx1 activates the transcription of miR-31, and miR-31 suppresses *Alx1* (Figure 4B). However, we were not able to bioinformatically identify potential Alx1 binding site upstream of *Sp*miR-31 and *Lv*miR-31 (Table 1). This may be due to indirect regulation of miR-31 by Alx1, or may represent a predictive failure where the inferred binding specificity based on available data from human and murine Alx1 does not match the binding specificity of *Sp*Alx1, as a result of evolutionary changes to the protein acquired in echinoderms (Lynch and Wagner, 2008; Khor and Ettensohn, 2017; Shashikant et al., 2018). Overall, our results strongly suggest that Alx1 and miR-31 participate in a possible regulatory loop that impacts sea urchin skeletogenesis (Figures 3, 4B) (Stepicheva and Song, 2015).

Worth noting is that our results indicate that knockdown of Ets1 results in a significant increase in miR-71 levels (Figures 3, 4C). miR-71 is an invertebrate-specific miRNA with known roles in L/R axis specification, olfactory neuron function, and aging in *C. elegans* (Boulias and Horvitz, 2012; Hsieh et al., 2012)*.* miR-71 is also necessary for survival of primary cells in *E. multilocularis,* and oogenesis in *L. migratoria* (Lucanic et al., 2013; Finger et al., 2019; Pérez et al., 2019; Song et al., 2019). It is additionally present in the roundworm *Brugia malayi*, and is involved in helminth parasitism (Liu C. et al., 2015; Rojas-Pirela et al., 2022). Ets1 is a highly evolutionarily conserved transcription factor, which in vertebrates is involved in development of melanocytes and the coronary vascular endothelium, as well as organ formation from mesodermal cells (Kola et al., 1993; Saldana-Caboverde et al., 2015; Wang et al., 2022). In echinoderms, Ets1 is involved in skeletogenesis alongside Alx1 (Kurokawa et al., 1999; 1999; Ettensohn et al., 2003; Rizzo et al., 2006; Oliveri et al., 2008; Yajima et al., 2010). We do not currently know the function of *Sp*miR-71. Further research into miR-71 may lead to discovery of conserved roles in echinoderm neurogenesis and skeletogenesis.

Our prior research has shown that *Sp*miR-124 directly suppresses *Notch* to regulate neural development and SMC differentiation (Konrad and Song, 2022; Konrad et al., 2023). Bioinformatic results from this study indicate that *Sp*miR-124 may be regulated by transcription factors downstream of Delta/Notch signaling (Table 1), suggesting a possible cross-regulatory relationship during these processes (Figure 4E).

Computational predictions were one of the lines of evidence we used to identify possible regulatory relationships governing level of miRNAs, and analysis of those predictions requires context in order to evaluate results thoroughly and draw reasonable conclusions. Even with a strong *p*-value for a given TF binding prediction, we have to take other factors into account. For example, inherent biological variability exists in both the binding sites recognized by a given TF, and conversely, the variety of TFs that can bind to a given short DNA sequence (Todeschini et al., 2014; Kribelbauer et al., 2019). CIS-BP, the data base we used for TF binding information, creates inferred sequence-binding motifs based on available evidence, sometimes in organisms that have large evolutionary distance from the sea urchin. In the case of Alx1 and Tbr, they may have acquired changes to their coding sequences over evolutionary time resulting in different binding specificities in echinoderms compared to vertebrates (Lynch and Wagner, 2008; Cheatle Jarvela et al., 2014; Cary et al., 2017; Shashikant et al., 2018). While TFs can retain DNA-binding specificity over evolutionary distance and through significant changes in sequence, orthologous transcription factors can obtain new activities and lose others in different species (Hanks et al., 1998; Siegal and Baker, 2005; Lynch and Wagner, 2008). An additional caveat to consider is that the statistical significance of individual motif occurrences alone is not directly comparable across all transcription factors. The length and complexity of the binding motif affects the “baseline” *p*-value for a given occurrence, and these qualifiers vary widely among different transcription factors. For example, the consensus binding motifs for FoxC1 and other Forkhead TFs tend to be shorter (∼10nt) and A-rich, and the binding motif for Klf15 is highly C-rich, resulting in numerous hits for these factors scattered across lower-complexity stretches of sequence in the scanned region (Weirauch et al., 2014). Furthermore, sequence-based predictions do not necessarily correlate with other lines of evidence, such as in *Sp*miR-1, where 10 Kruppel-like factor 15 binding sites are predicted, but only four overlap with ATAC-seq (Table 1). Given that sequence specificity alone may not prove conclusive, we used various additional lines of evidence to strengthen the confidence derived from a predicted TF binding sites. This includes agreement with our own experimental data, where we demonstrate that levels of some miRNAs were modulated by predicted TFs downstream of a particular targeted signaling pathway. Also, the genome resource site for echinoderms, the Echinobase, provides ATAC-seq data across multiple timepoints of embryonic development (Arshinoff et al., 2022; Telmer et al., 2024). ATAC-seq data yields alignments to the genome wherever DNA is not closed off by chromatin, and is thus open to TF binding and transcription (Buenrostro et al., 2013; Buenrostro et al., 2015; Shashikant et al., 2018; Shashikant and Ettensohn, 2019). Any region of DNA which is accessible to ATAC-seq is potentially accessible for TF binding, and *vice versa*. Thus, we use the ATAC-Seq data to evaluate the possible functionality of a TF at a predicted binding site at any given stage in development.

Another criterion that we use to analyze our results is conservation of sequence in the proposed regulatory region across species (Brown et al., 2005; Rebeiz et al., 2015). For example, we identified a shared occurrence of caudal type homeobox 1-like binding sites upstream of *Sp*miR-2012 and *Lv*miR-2012 (Table 1). In general, we find the majority of TFs predicted in the purple sea urchin to be corroborated in the green sea urchin. In *Sp*miR-1, *Sp*miR-31, and *Sp*miR-124, 100% of predictions were corroborated with *Lv*miR-1, *Lv*miR-31, and *Lv*miR-124, respectively (Table 1, Supplementary Table S3). In general, using a cross-species evolutionary analysis approach, our results indicate that 73% of TF sequences found in S. *purpuratus* miRNA loci are also predicted in the corresponding *L. variegatus* miRNA loci (Table 1, Supplementary Table S3).

Proximity of the predicted binding site to the miRNA is an additional consideration. It has been shown that regions proximal to a given transcription start site (TSS) are more likely to contain TF binding sites (Lin et al., 2010; Whitfield et al., 2012). Considering this, the distance of a predicted binding motif from the miRNA itself can also serve as evidence for or against the validity of that prediction. For example, in the case of the Kruppel like factor 15, SNAI1, and HMG protein Tcf/Lef, each is predicted to bind within 500 bp upstream of *Sp*miR-1*,* with Tcf/Lef binding within 1000 bp in *Lv*miR-1 (Table 1).

In summary, significant changes in levels of multiple miRNAs were observed upon disruption of signaling pathways and transcription factors. We have identified several instances where miRNA level is dependent on developmental signaling pathways. The fundamental goal of the computational analysis performed was to identify the specific TFs which mediate the regulation of miRNA expression demonstrated in the experimental data presented. Our results provide multiple lines of evidence to propose reasonable TFs downstream of specific signaling pathways that may regulate miRNA levels (Figure 4). However, to definitively assess direct regulation of these TFs of specific miRNA, experimental testing will be required.

Overall, this study provides a deeper insight and understanding of how miRNAs are transcriptionally regulated by signaling pathways and transcription factors during embryogenesis. Since post-transcriptional regulation mediated by miRNAs is a key regulator of development, alongside signaling pathways and transcription factors, a greater understanding of how they regulate and cross-regulate contributes to our overall understanding of development.

## Data Availability

The datasets presented in this study can be found in online repositories. The names of the repository/repositories and accession number(s) can be found in the article/Supplementary Material.

## Supplementary material

SUPPLEMENTARY FIGURE S1 Perturbations of cadherin and TFs result in expected defects. **(A)** Truncated cadherin was injected at 300 ng/µl into newly fertilized eggs. Embryos are animalized with no endomesodermally-derived structures. The red arrow indicates the PMCs. Lateral view of blastulae is shown. All scale bars are 50 µm. **(B)** 2 mM of Alx1 MASO, 2 mM of Ets1 MASO, and 0.7 mM Tbr MASO were injected into newly fertilized eggs. Ets1 and Alx1 MASO resulted in no PMCs; Tbr MASO resulted in no skeleton. The red arrows indicate the skeletal spicules in the gastrulae in lateral view.

SUPPLEMENTARY FIGURE S2 Inhibition of various signaling pathways did not result in changes of miRNA levels. Sea urchin zygotes were treated with inhibitors of various signaling pathways. Inhibitors of Delta/Notch, VEGF, BMP, and Nodal signaling pathways were tested. This was followed by qPCR against miRNAs. No significant changes of miRNA levels were observed.

SUPPLEMENTARY FIGURE S3 Schematic comparison of miRNA genomic regions in S. purpuratus and L. variegatus. The Echinobase genome browser was used to identify genes adjacent to miRNA loci in S. purpuratus and L. variegatus genomes to evaluate evolutionary conservation of genes (Arshinoff et al., 2022; Telmer et al., 2024). Diagram not to scale.

SUPPLEMENTARY FIGURE S4 Genomic region surrounding miR-1 is evolutionarily conserved between echinoderms and mammals. **(A)** Comparison of NCBI genome browser view of regions surrounding miR-1 in human and mouse against schematics of the orthologous region in the purple and green sea urchins. In all species, miR-1 and miR-133 are nested within a region that is either an intron of the gene Mib1, or an intergenic region between two separate Mib1 transcripts. **(B)** PCR primers spanning the Mib1 and SpmiR-1 regions were designed to resolve genomic structure of SpmiR-1. PCR shows a single Mib1 transcript encompassing SpmiR-1 locus. Total RNA from 24hpf S. purpuratus embryos was used to generate cDNA which was used as a template for PCR. Control primers against Mib1 exons on either side of miR-1 produced correct expected sizes. The experimental set of primers was designed to span exons on either side of the region containing SpmiR-1. There is a ∼142 kb span between the nearest exons on either side of SpmiR-1, if this region were intergenic, the PCR product would be too large to amplify. However, if the two genes annotated as “Spmib1” on either side of SpmiR-1 were in fact a single transcript, the ∼140 kb span would be processed as an intron, with the remaining coding sequences short enough to amplify and analyze by PCR of embryo cDNA. The results indicated a PCR product of 400 bp, indicating that miR-1 is likely in the intronic region of Mib1. Primers from 5’ to 3’ are the following: ExperimentFor: GCT​AAA​TGA​AGG​GCC​GAC​TG; ExperimentRev: CCA​CCC​AAG​TCT​CCA​GGA​TT; Control1For: GCT​AAA​TGA​AGG​GCC​GAC​TG; Control1Rev: TTA​TAC​CGC​CCT​CCA​TCT​CG; Control2For: TGT​CAA​AAT​CAG​GGG​TGC​AG; Control2Rev: GGG​GTC​AAC​AAG​GGT​CAC​TA.
