# Supplementary figures and images for: Transcription of microRNAs is regulated by developmental signaling pathways and transcription factors
Source: Front Cell Dev Biol. 2024 Apr 24;12:1356589. doi: 10.3389/fcell.2024.1356589 (PMC11076791; doi:10.3389/fcell.2024.1356589)

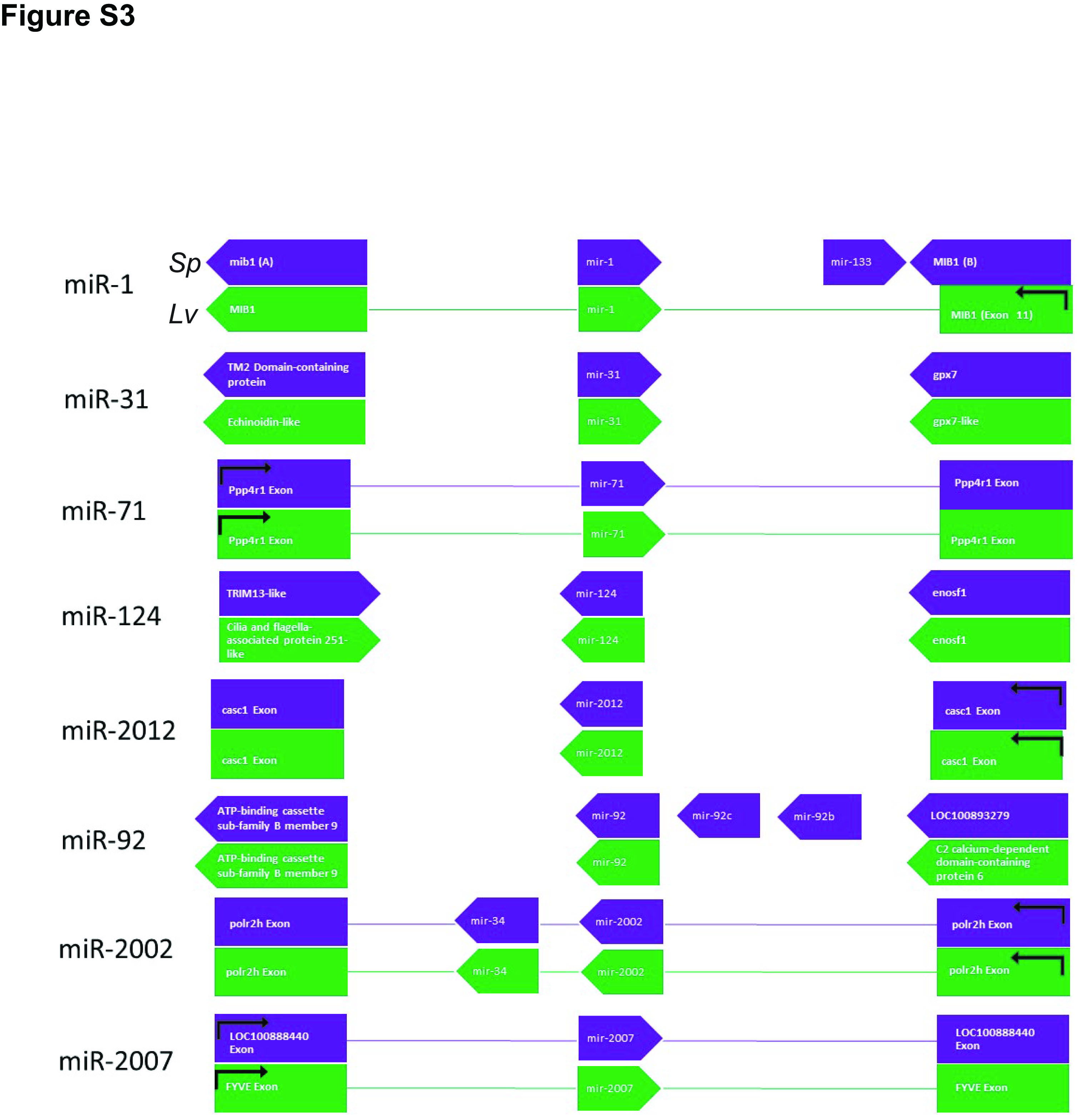

Supplement: Supplementary file 1 [file Image3.jpeg]

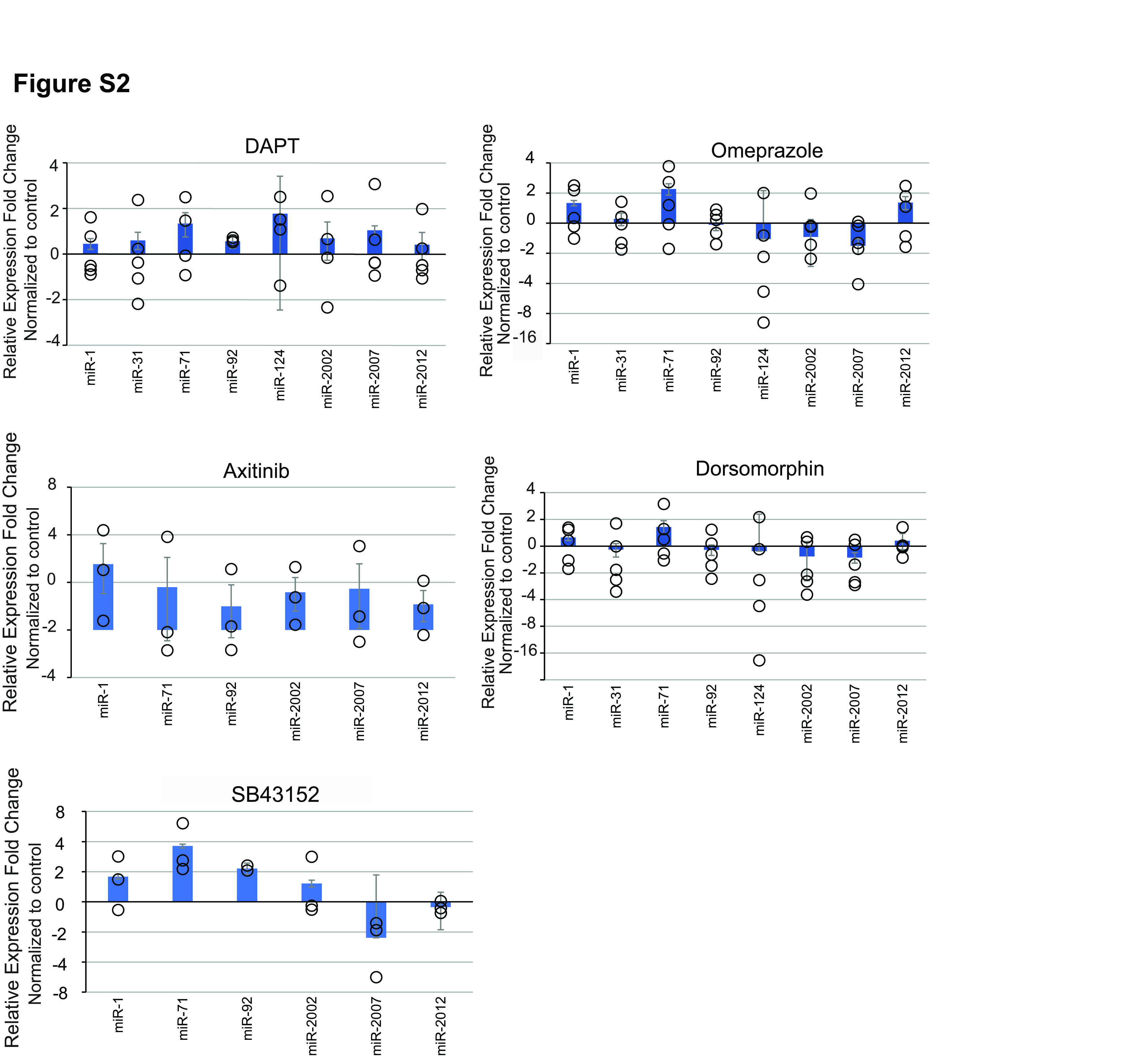

Supplement: Supplementary file 3 [file Image2.jpeg]

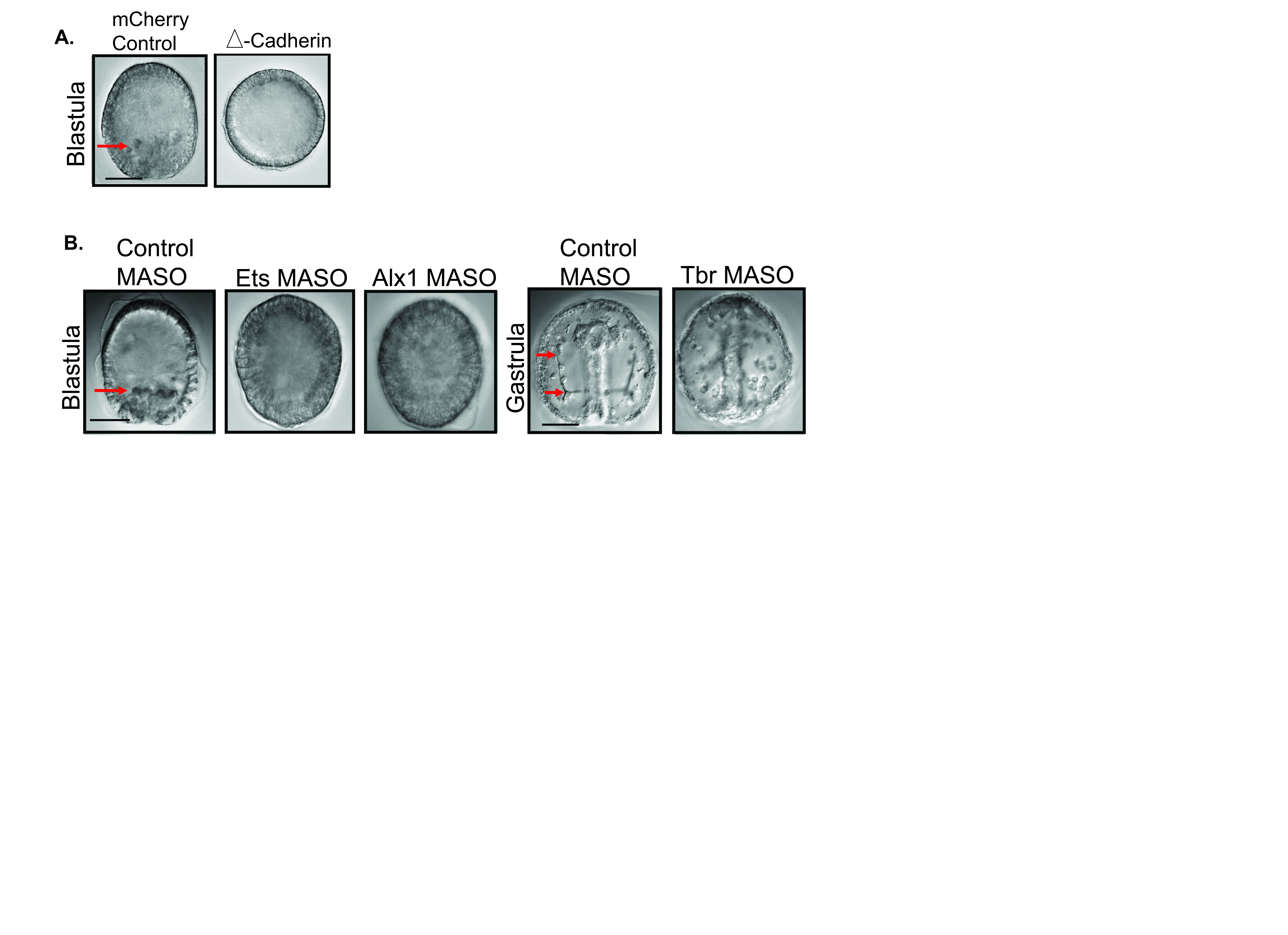

Supplement: Supplementary file 4 [file Image1.tif]

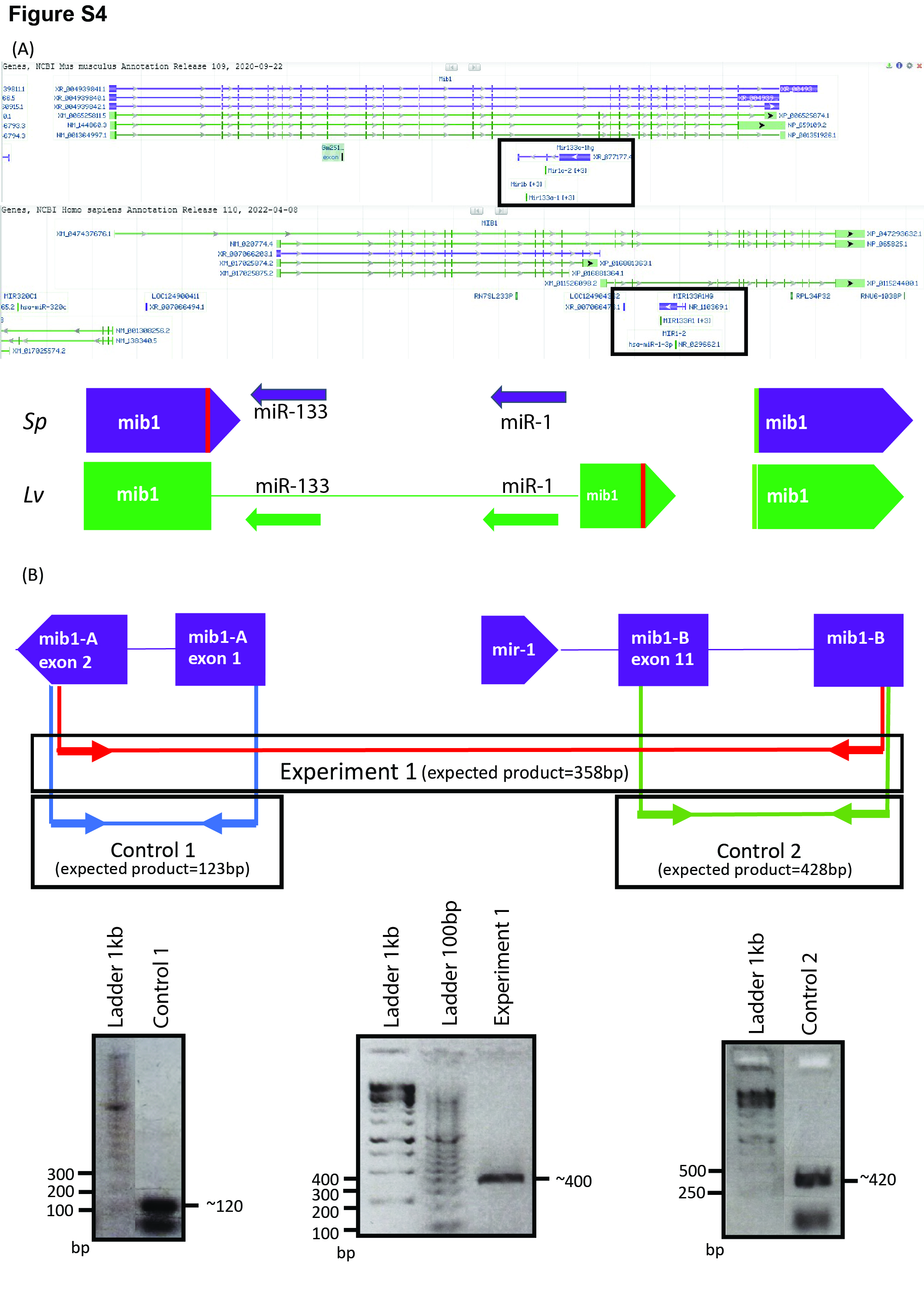

Supplement: Supplementary file 7 [file Image4.jpg]
