# Supplementary material for: Transcription of microRNAs is regulated by developmental signaling pathways and transcription factors
Source: Front Cell Dev Biol. 2024 Apr 24;12:1356589. doi: 10.3389/fcell.2024.1356589 (PMC11076791; doi:10.3389/fcell.2024.1356589)
Supplement: Supplementary file 2 [file Table1.docx]

**Table S1 Pharmaceutical Inhibitors Against Signaling Pathways**

| **Inhibitor** | **Pathway** | **Concentration** | **References** | **Outcomes** |
| --- | --- | --- | --- | --- |
| Axitinib | VEGFR signaling | 50nM | (Adomako-Ankomah and Ettensohn, 2013) | PMC and skeletogenesis inhibited |
| C59 | All Wnt branches | 0.5µM | (Cui et al., 2014) | Gastrulation defects |
| U0126 | MAPK/ERK | 0.1µM | (Kumano and Foltz, 2003; Rottinger et al., 2004) | Prevents PMC ingression & lack skeleton |
| Y-27632 | ROCK (ncWnt/PCP) | 50µM | (Beane et al., 2006; Croce et al., 2006) | Lack of skeleton |
| Bisindolylmaleimide I | PKC (ncWnt/PCP) | 2.5 µM | (Croce et al., 2006) | Skeletogenesis inhibited |
| SB431542 | Nodal | 1 µM | (Piacentino et al., 2015; Sun and Ettensohn, 2017) | Loss of left/right asymmetry. Nodal expression abolished. |
| A-83-01 | Nodal | 2 µM | (Tojo et al., 2005) | Loss of left/right asymmetry. |
| SP600125 | JNK (ncWnt/PCP) | 0.15 µM | (Croce et al., 2006; Long et al., 2015) | Lack of an archenteron. Exogastrulation |
| ML141 | Cdc42 (ncWnt/PCP) | 2.5 µM | (Sepulveda-Ramirez et al., 2018) | Skeletal defects and developmental delay |
| Dorsomorphin | BMP | 10 µM | (Luo and Su, 2012) | Loss of left/right asymmetry. |
| Cyclopamine | Sonic hedgehog | 1 µM | (Warner and McClay, 2014) | Truncated skeleton and decreased pigment cells |
| DAPT | Delta/Notch | 0.25 µM | (Materna and Davidson, 2012) | Lack of coelomic pouches, no pigment cells |
| Omeprazole | Delta/Notch | 100 µM | (Bessodes et al., 2012) | Delays gastrulation and lacked pigment cells |
