# Supplementary material for: Transcription of microRNAs is regulated by developmental signaling pathways and transcription factors
Source: Front Cell Dev Biol. 2024 Apr 24;12:1356589. doi: 10.3389/fcell.2024.1356589 (PMC11076791; doi:10.3389/fcell.2024.1356589)
Supplement: Supplementary file 5 [file Table2.docx]

**Table S2 MicroRNA RT-qPCR Primers**

| **microRNA** | **(5’ to 3’)**  **qPCR primer sequence of mature miRNA** | **Qiagen catalog number** |
| --- | --- | --- |
| **Spu-miR-1** | Hsa-miR-1-3p UGGAAUGUAAAGAAGUAUGUAU | YP00204344 |
| **Spu-miR-31** | Spu-miR-31  AGGCAAGAUGUUGGCAUAGCU-- | YP02111135 |
| **Spu-miR-71** | Spu-miR-71  UGAAAGACAUGGGUAGUGAGAUU | YP02107666 |
| Spu-miR-92e | Spu-miR-92e  UAUUGCACUUACCCCGGCUUA | YP02104564 |
| Spu-miR-124 | Cbr-miR-124a  UAAGGCACGCGGUGAAUGCCA- | YP02103368 |
| Spu-miR-200-3p | Spu-miR-200-3p  UAAUACUGUCUGGUGAUGAUGUU | YP02112491 |
| Spu-miR-2002-3p | Spu-miR-2002-3p  UGAAUACAUCUGCUGGUUUUUAU | YP02110710 |
| Spu-miR-2007 | Spu-miR-2007  UAUUUCAGGCAGUAUACUGGUA | YP02105686 |
| **Spu-miR-2012** | Sko-miR-2012-5p  UAGUACUGGCAUAUGGACAUUG | YP02106647 |

# Most highly expressed miRNAs in *S. purpuratus* are **bolded;** Seed region is underlined
