# Supplementary material for: Transcription of microRNAs is regulated by developmental signaling pathways and transcription factors
Source: Front Cell Dev Biol. 2024 Apr 24;12:1356589. doi: 10.3389/fcell.2024.1356589 (PMC11076791; doi:10.3389/fcell.2024.1356589)
Supplement: Supplementary file 6 [file Table3.docx]

**Table S3: Processed FIMO Results for miRNAs in *S. purpuratus* (Sp) and *L. variegatus* (Lv)**

| miRNA | Gene Name | Motif Type | Regulated by: | Up vs. Down Regulation | p-value | q-value* | Distance from miRNA | Overlap with ATAC-seq | References |
| --- | --- | --- | --- | --- | --- | --- | --- | --- | --- |
| *Sp*-miR-1 |  |  |  |  |  |  |  |  |  |
| *Lv*-miR-1 | lin-54 DREAM MuvB core complex component | TCR/CxC_direct_M02531_2.00 | Wnt | Up | 5.45E-05 | 0.313 | 3824, 9919 | All | (Cherian et al., 2020) |
|  | T-box transcription factor 2 | M08044_2.00 | cWnt, TGF-β, FGF | Up, Down, Up | 3.64E-05 | 0.363 | 2815, 8660 | MC, LC, HB, MG, EL | (Firnberg and Neubüser, 2002; Li et al., 2014; Lüdtke et al., 2016; Aydoğdu et al., 2018) |
|  | hepatocyte nuclear factor 4 alpha | Nuclear_receptor_  inferred_M08219_2.00 | cWnt | Down | 6.47E-05 | 0.412 | 751, 9921 | MC, LC, HB, MG, EL | (Yang et al., 2013) |
|  | paired class homeodomain repressor | M00291_2.00 | cWnt | Up | 3.17E-05 | 0.245 | 8191 | All | (Cavalieri et al., 2017) |
| *Sp-*miR-31 | YY1 transcription factor | C2H2 ZF | TGF-β | Up | 5.89E-06 | 0.13 | 1284 | None | (Zhang et al., 2019) |
| *Lv*-miR-31 | Kruppel like factor 6 | C2H2_ZF_inferred_M08298_2.00 | TGF-β /Smad3 | Up | 7.70E-05 | 0.17 | 5443, 9085, 9199 | EC, MC | (Dionyssiou et al., 2013) |
|  | homeobox protein MIXL1-like | M03187_2.00 | TGF-β, Nodal | Up | 3.73E-05 | 0.26 | 934, 2633, 5418, 6079 | MC, LC, HB, MG, EL | (Choi et al., 2015) |
|  | YY1 transcription factor | M08288_2.00 |  | Up | 7.83E-05 | 1 | 1299 | None | (Zhang et al., 2019) |
| *Sp-*miR-71 | snail family transcriptional repressor 1 (SNAI1) | C2H2 ZF | Wnt, SHH | Up | 3.96E-05 | 0.541 | 469 | 18, 24, 30, 39, 50, 60 hrs | (Horvay et al., 2011; Heiden et al., 2014) |
|  | hepatocyte nuclear factor 4 alpha | Nuclear receptor | Wnt | Down | 1.57E-05 | 0.305 | 2494 | 18, 24, 30, 39, 50, 60, 70 hrs | (Yang et al., 2013) |
|  | B-cell lymphoma/leukemia 11A | C2H2 ZF | ncWnt | Down | 8.66E-06 | 0.082 | 7786 | 24, 30, 39, 50, 60 hrs | (Xiao et al., 2016) |
| *Lv*-miR-71 | caudal type homeobox 1-like | M08131_2.00 | cWnt | Up | 9.67E-05 | 0.24 | 4143, 4180, 4767, 5848, 6608 | None | (Lickert et al., 2000) |
|  | paired class homeodomain repressor | M00291_2.00 | cWnt | Up | 2.28E-05 | 0.401 | 9404, 9884 | All | (Cavalieri et al., 2017) |
|  | gastrulation brain homeobox 2-like | M03847_2.00 | Wnt, BMP | Up | 6.52E-05 | 1 | 7108 | All | (Li et al., 2009) |
|  | HMG protein Tcf/Lef | M08167_2.00 | Wnt | Up | 5.00E-06 | 0.0831 | 877, 7026, 7231, 7573, 9825 | MC, LC, HB, MG, EL | (Novak and Dedhar, 1999) |
|  | lin-54 DREAM MuvB core complex component | TCR/CxC_direct_M02531_2.00 | Wnt | Up | 4.81E-05 | 0.154 | 3135, 5502, 6647, 8870 | MC, LC, HB, EL | (Cherian et al., 2020) |
|  | T-box transcription factor 2 | M08044_2.00 | cWnt, TGF-β, FGF | Up, Down, Up | 1.24E-05 | 0.247 | 2105 | MC, LC, HB, MG, EL | (Firnberg and Neubüser, 2002; Li et al., 2014; Lüdtke et al., 2016; Aydoğdu et al., 2018) |
| *Sp*-miR-124 |  |  |  |  |  |  |  |  |  |
| *Lv*-miR-124 | achaete-scute family bHLH transcription factor 1 | M08055_2.00 | Delta/Notch | Down | 3.93E-05 | 0.79 | 9461 | MC, LC, HB, MG | (Hibdon et al., 2023) |
|  | B-cell lymphoma/leukemia 11A | M07926_2.00 | ncWnt | Down | 4.86E-05 | 0.264 | 1491, 2506, 4829, 7212, 7243 | MC, LC, HB, EL | (Xiao et al., 2016) |
|  | gastrulation brain homeobox 2-like | M03847_2.00 | Wnt, BMP | Up | 4.69E-05 | 0.815 | 2533 | EC, MC, LC, MG | (Li et al., 2009) |
|  | HMG protein Tcf/Lef | M08167_2.00 | Wnt | Up | 9.70E-05 | 0.619 | 2257, 9540 | EC, MC, LC, HB, EL | (Novak and Dedhar, 1999) |
|  | homeobrain | M03813_2.00 | ncWnt |  | 6.30E-05 | 0.437 | 6823, 7201 | MC, LC, HB, MG | (Yaguchi et al., 2016) |
|  | Kruppel like factor 6 | C2H2_ZF_inferred_M08298_2.00 | TGF-β /Smad3 | Up | 1.14E-05 | 0.23 | 1821 | MG, EL | (Dionyssiou et al., 2013) |
|  | lin-54 DREAM MuvB core complex component | TCR/CxC_direct_M02531_2.00 | Wnt | Up | 9.07E-05 | 0.151 | 1634, 3164, 7791, 8785 | LC, MG | (Cherian et al., 2020) |
|  | myocyte enhancer factor 2C-like | MADS_box_inferred_M08149_2.00 | IGF1,MAPK,Delta/Notch,Snail1,Wnt,B-catenin | Β-catenin down, all else Up | 5.48E-05 | 0.498 | 3233, 6128 | EC, MC, LC | (Khiem et al., 2008; Muñoz et al., 2009; Bai et al., 2015; Ignatius et al., 2017) |
|  | SIX homeobox 4 | M00461_2.00 | Tcf/Lef (Wnt) | Up | 7.72E-05 | 0.776 | 2637, 9458 | MG, EL | (Sato et al., 2015) |
|  | transcription factor MafK-like | M02854_2.00 | Wnt | Up | 1.68E-05 | 0.195 | 1280 | EC | (Wang et al., 2015) |
|  | YY1 transcription factor | M08288_2.00 | TGF-β | Up | 4.40E-06 | 0.0442 | 3116, 6436 | MG | (Zhang et al., 2019) |
| *Sp-*miR-2007 | YY1 transcription factor | M08288_2.00 | TGF-β | Up | 3.49E-06 | 0.0626 | 1686 | None | (Zhang et al., 2019) |
|  | Kruppel like factor 6 | kruppel_like_factor_6_M08298_2.00 | TGF-β /Smad3 | Up | 7.03E-06 | 0.14 | 4551 | None | (Dionyssiou et al., 2013) |
| *Lv-*miR-2007 |  |  |  |  |  |  |  |  |  |
| *Sp-*miR-2012 | Kruppel like factor 15 | C2H2 ZF | Wnt | Down | 8.46E-07 | 0.00168 | 5620 | None | (Noack et al., 2019) |
| *Lv-*miR-2012 | forkhead box C1 | Forkhead_inferred_M00257_2.00 | TGF-β | Up | 6.76E-05 | 0.15 | 274, 3314, 4693, 5431 | All | (Massagué, 1998; Zhang et al., 2019) |
|  | lin-54 DREAM MuvB core complex component | TCR/CxC_direct_M02531_2.00 | Wnt | Up | 2.00E-06 | 0.0169 | 416, 2806, 3552, 6040, 6073, 6414 | All | (Cherian et al., 2020) |
|  | myocyte enhancer factor 2C-like | MADS_box_inferred_M08149_2.00 | IGF1, MAPK,Delta/Notch,SnaiL1,Wnt,B-catenin | Β-catenin down, all else Up | 9.43E-05 | 0.756 | 2254, 6909 | All | (Khiem et al., 2008; Muñoz et al., 2009; Bai et al., 2015; Ignatius et al., 2017) |
|  | T-box transcription factor 2 | M08044_2.00 | cWnt, TGF-β, FGF | Up, Down, Up | 7.67E-05 | 1 | 7987 | All | (Firnberg and Neubüser, 2002; Li et al., 2014; Lüdtke et al., 2016; Aydoğdu et al., 2018) |
|  | transcription factor MafK-like | M02854_2.00 | Wnt | Up | 2.03E-05 | 0.108 | 308, 4558, 7055 | All | (Wang et al., 2015) |

*The q-value measures false positive rate(Storey and Tibshirani, 2003).

Aydoğdu, N., Rudat, C., Trowe, M.-O., Kaiser, M., Lüdtke, T. H., Taketo, M. M., et al. (2018). TBX2 and TBX3 act downstream of canonical WNT signaling in patterning and differentiation of the mouse ureteric mesenchyme. *Development* 145, dev171827. doi: 10.1242/dev.171827

Bai, X. L., Zhang, Q., Ye, L. Y., Liang, F., Sun, X., Chen, Y., et al. (2015). Myocyte enhancer factor 2C regulation of hepatocellular carcinoma via vascular endothelial growth factor and Wnt/β-catenin signaling. *Oncogene* 34, 4089–4097. doi: 10.1038/onc.2014.337

Cavalieri, V., Geraci, F., and Spinelli, G. (2017). Diversification of spatiotemporal expression and copy number variation of the echinoid hbox12/pmar1/micro1 multigene family. *PLoS ONE* 12, e0174404. doi: 10.1371/journal.pone.0174404

Cherian, J. R., Adams, K. V., and Petrella, L. N. (2020). Wnt Signaling Drives Ectopic Gene Expression and Larval Arrest in the Absence of the *Caenorhabditis elegans* DREAM Repressor Complex. *G3 Genes|Genomes|Genetics* 10, 863–874. doi: 10.1534/g3.119.400850

Choi, S.-C., Choi, J.-H., Cui, L.-H., Seo, H.-R., Kim, J.-H., Park, C.-Y., et al. (2015). Mixl1 and Flk1 Are Key Players of Wnt/TGF-β Signaling During DMSO-Induced Mesodermal Specification in P19 cells: DMSO-INDUCED MEDOSERMAL DIFFERENTIATION OF P19 CELLS. *J. Cell. Physiol.* 230, 1807–1821. doi: 10.1002/jcp.24892

Dionyssiou, M. G., Salma, J., Bevzyuk, M., Wales, S., Zakharyan, L., and McDermott, J. C. (2013). Krüppel-like factor 6 (KLF6) promotes cell proliferation in skeletal myoblasts in response to TGFβ/Smad3 signaling. *Skeletal Muscle* 3, 7. doi: 10.1186/2044-5040-3-7

Firnberg, N., and Neubüser, A. (2002). FGF signaling regulates expression of Tbx2, Erm, Pea3, and Pax3 in the early nasal region. *Dev Biol* 247, 237–250. doi: 10.1006/dbio.2002.0696

Heiden, K. B., Williamson, A. J., Doscas, M. E., Ye, J., Wang, Y., Liu, D., et al. (2014). The sonic hedgehog signaling pathway maintains the cancer stem cell self-renewal of anaplastic thyroid cancer by inducing snail expression. *J Clin Endocrinol Metab* 99, E2178-2187. doi: 10.1210/jc.2014-1844

Hibdon, E. S., Keeley, T. M., Merchant, J. L., and Samuelson, L. C. (2023). The bHLH Transcription Factor ASCL1 Promotes Differentiation of Endocrine Cells in the Stomach and is Regulated by Notch Signaling. *American Journal of Physiology-Gastrointestinal and Liver Physiology*, ajpgi.00043.2023. doi: 10.1152/ajpgi.00043.2023

Horvay, K., Casagranda, F., Gany, A., Hime, G. R., and Abud, H. E. (2011). Wnt Signaling Regulates Snai1 Expression and Cellular Localization in the Mouse Intestinal Epithelial Stem Cell Niche. *Stem Cells and Development* 20, 737–745. doi: 10.1089/scd.2010.0188

Ignatius, M. S., Hayes, M. N., Lobbardi, R., Chen, E. Y., McCarthy, K. M., Sreenivas, P., et al. (2017). The NOTCH1/SNAIL1/MEF2C Pathway Regulates Growth and Self-Renewal in Embryonal Rhabdomyosarcoma. *Cell Reports* 19, 2304–2318. doi: 10.1016/j.celrep.2017.05.061

Khiem, D., Cyster, J. G., Schwarz, J. J., and Black, B. L. (2008). A p38 MAPK-MEF2C pathway regulates B-cell proliferation. *Proc. Natl. Acad. Sci. U.S.A.* 105, 17067–17072. doi: 10.1073/pnas.0804868105

Li, B., Kuriyama, S., Moreno, M., and Mayor, R. (2009). The posteriorizing gene *Gbx2* is a direct target of Wnt signalling and the earliest factor in neural crest induction. *Development* 136, 3267–3278. doi: 10.1242/dev.036954

Li, J., Ballim, D., Rodriguez, M., Cui, R., Goding, C. R., Teng, H., et al. (2014). The Anti-proliferative Function of the TGF-β1 Signaling Pathway Involves the Repression of the Oncogenic TBX2 by Its Homologue TBX3. *Journal of Biological Chemistry* 289, 35633–35643. doi: 10.1074/jbc.M114.596411

Lickert, H., Domon, C., Huls, G., Wehrle, C., Duluc, I., Clevers, H., et al. (2000). Wnt/β-catenin signaling regulates the expression of the homeobox gene *Cdx1* in embryonic intestine. *Development* 127, 3805–3813. doi: 10.1242/dev.127.17.3805

Lüdtke, T. H., Rudat, C., Wojahn, I., Weiss, A.-C., Kleppa, M.-J., Kurz, J., et al. (2016). Tbx2 and Tbx3 Act Downstream of Shh to Maintain Canonical Wnt Signaling during Branching Morphogenesis of the Murine Lung. *Dev Cell* 39, 239–253. doi: 10.1016/j.devcel.2016.08.007

Massagué, J. (1998). TGF-β SIGNAL TRANSDUCTION. *Annu. Rev. Biochem.* 67, 753–791. doi: 10.1146/annurev.biochem.67.1.753

Muñoz, J. P., Collao, A., Chiong, M., Maldonado, C., Adasme, T., Carrasco, L., et al. (2009). The transcription factor MEF2C mediates cardiomyocyte hypertrophy induced by IGF-1 signaling. *Biochemical and Biophysical Research Communications* 388, 155–160. doi: 10.1016/j.bbrc.2009.07.147

Noack, C., Iyer, L. M., Liaw, N. Y., Schoger, E., Khadjeh, S., Wagner, E., et al. (2019). KLF15-Wnt–Dependent Cardiac Reprogramming Up-Regulates SHISA3 in the Mammalian Heart. *Journal of the American College of Cardiology* 74, 1804–1819. doi: 10.1016/j.jacc.2019.07.076

Novak, A., and Dedhar, S. (1999). Signaling through ?-catenin and Lef/Tcf. *Cellular and Molecular Life Sciences (CMLS)* 56, 523–537. doi: 10.1007/s000180050449

Sato, S., Yajima, H., Furuta, Y., Ikeda, K., and Kawakami, K. (2015). Activation of Six1 Expression in Vertebrate Sensory Neurons. *PLoS ONE* 10, e0136666. doi: 10.1371/journal.pone.0136666

Wang, R., Zheng, J., Zhang, D.-S., Yang, Y.-H., and Zhao, Z.-F. (2015). Wnt1-induced MAFK expression promotes osteosarcoma cell proliferation. *Genet. Mol. Res.* 14, 7315–7325. doi: 10.4238/2015.July.3.7

Xiao, J., Zhou, H., Wu, N., and Wu, L. (2016). The non-canonical Wnt pathway negatively regulates dendritic cell differentiation by inhibiting the expansion of Flt3+ lymphocyte-primed multipotent precursors. *Cell Mol Immunol* 13, 593–604. doi: 10.1038/cmi.2015.39

Yaguchi, J., Takeda, N., Inaba, K., and Yaguchi, S. (2016). Cooperative Wnt-Nodal Signals Regulate the Patterning of Anterior Neuroectoderm. *PLoS Genet* 12, e1006001. doi: 10.1371/journal.pgen.1006001

Yang, M., Li, S.-N., Anjum, K. M., Gui, L.-X., Zhu, S.-S., Liu, J., et al. (2013). Double-negative feedback loop between Wnt/β-catenin signaling and HNF4α regulates epithelial-mesenchymal transition in hepatocellular carcinoma. *Journal of Cell Science*, jcs.135053. doi: 10.1242/jcs.135053

Zhang, C., Zhu, X., Hua, Y., Zhao, Q., Wang, K., Zhen, L., et al. (2019). YY1 mediates TGF-β1-induced EMT and pro-fibrogenesis in alveolar epithelial cells. *Respir Res* 20, 249. doi: 10.1186/s12931-019-1223-7
